# Through Human Eyes: Owner Insights into the Social Relationships of Pet Rats

**DOI:** 10.3390/ani15172579

**Published:** 2025-09-02

**Authors:** Caitlin Walburn, Emily Blackwell, Mike Mendl, Elizabeth S. Paul, Vikki Neville

**Affiliations:** Bristol Veterinary School, University of Bristol, Bristol BS40 5DU, UK

**Keywords:** rat, social behaviour, companion animal, reflective thematic analysis, animal welfare, human–animal interaction, human–animal bond

## Abstract

Understanding the social relationships and behaviours of pet rats is important, particularly because they are comparatively understudied compared to their laboratory and wild counterparts, and little is known about their welfare. Here, open-ended interviews, with a particular focus on rat social relationships and behaviours, were conducted with 23 pet rat owners in the United Kingdom and a reflective thematic analysis was conducted on the resulting, transcribed dataset. Seven main themes were generated: Social Behaviours, Social Life and Group Dynamics, Introducing New Rats and Repairing Social Bonds, Owner Practices, Participant and Rat Contextual Background, Owner Narratives and Shared Understandings, and Owner Research Interests. Owners described rat social relationships and behaviours with a high level of consistency and reported the techniques they employ to manage the social dynamics of their rat groups, including the first scientific report of rat introductions. We propose that these qualitative findings can inform future research, including observational studies of captive (pet and non-pet) rat management and welfare.

## Simple Summary

Owner reports of their pets’ behaviour and behavioural interactions can be informative in a number of ways. For example, owner perceptions of their animals’ behaviours can aid in ensuring that the animals’ welfare standards are kept high. While research into companion animal social behaviours is underway in more popular companion animals such as dogs and cats, less is known about more unconventional pet species, like the rat. Owner interviews are a useful first step in finding out about the range and frequencies of rat social behaviours and about people’s perceptions of them. We designed open-ended interviews to explore rat owners’ reports of pet rat social behaviours and dynamics in the United Kingdom. Using a reflective thematic analysis technique, we analysed these interviews and found that pet rat owners consistently reported a number of social relationships and behaviours, which they described in terms of distinct categories of generally positive and generally negative interactions. Owners also discussed their relationships with their pet rats, and the methods they employ when introducing new rats into an established group.

## 1. Introduction

Developing an understanding of the social relationships and social behaviours exhibited by pet (companion) rats, and the situations in which these arise, is an important goal of contemporary rat welfare research. While laboratory and wild rat social behaviour is fairly well-documented [1,2,3,4,5,6,7,8,9], the social lives of rats in a companion animal setting are far less studied. Compared to laboratory rats, for example, pet rats live in variable environments with differing group compositions, housing types, and human interactions. These differences mean that findings from laboratory and wild settings may not always apply to pet rats, making them a worthy subject of study in themselves. Pet rats may also have information to offer to our understanding of their captive (e.g., laboratory) counterparts, by revealing rarer or more idiosyncratic behaviours that have not previously been observed or studied.

We propose three main reasons why pet rat research is important. First, it is important for improving our understanding of rat social dynamics (and, thereby, welfare) in the various living conditions that pet rats regularly encounter (e.g., male only/female only/mixed-sex groups). Second, it is important for ethogram development and improvement, a key step in the development of behavioural assays of pet and laboratory rat welfare (e.g., across different housing and enrichment conditions, where the constraints and welfare consequences of captivity can be considerable [10]). Third, studying pet rats offers the opportunity to understand rat social behaviour and behavioural needs more broadly, which could extend across all contexts of rodent research.

In recent years, increasing numbers of studies have used owner-interview and questionnaire studies to investigate pet animals’ behaviour and welfare (e.g., dogs [11,12,13,14,15], cats [16,17,18], both dogs and cats [19,20], and pets more generally [21]). Many of these apply qualitative methods of analysis, such as thematic analyses (e.g., [13,15,17,19]) or mixed methods in which qualitative and quantitative approaches are combined (e.g., [12,14,20,21]). While lacking the rigor of more traditional observational and experimental methods, such studies offer researchers a number of advantages and opportunities: for example, the capacity to explore complex and rarer behaviours and situations. Moreover, interview and questionnaire techniques can also offer an additional perspective—that of the pet owner themself—thereby providing a valuable additional layer of insight that can complement that of the researcher. In the present context, of pet rat social behaviour and welfare, this is particularly important for understanding where welfare issues in pet rat populations might arise—for example, by understanding the frequency of potentially negative social interactions and understanding how well owners can identify them as such. In addition, as owners are key parts of their pets’ environments, their inferences and interpretations of behaviour are likely to feed back directly to the animals in the form of human–animal interactions and husbandry practices.

The present study was designed to investigate the range of behaviours and social interactions shown by pet rats, as witnessed through the eyes of rat owners themselves. In particular, we aimed to explore owners’ perceptions of pet rat social relationships, focusing on how rat groups are composed, maintained and managed and to investigate which behaviours pet rat owners identified as ‘social’, in what contexts these were seen, and which of these behaviours they perceived to be either ‘positive’ (friendly, affiliative, etc.) or ‘negative’ (unfriendly, agonistic, etc.).

### 1.1. Rat Social Relationships and Behaviour

Rats have complex social systems and a variety of wild and laboratory studies have found them to exhibit a range of social behaviours [3,4,22]. For example, drawing on research conducted both in wild and laboratory settings, Schweinfurth, (2020) proposed an ethogram of rat social behaviours that was split into behaviours that are ‘socio-positive’ (e.g., allogrooming, play fighting, and sharing food) and ‘socio-negative’ (e.g., aggressive grooming, chasing, and fighting) [3]. It is thought that poor welfare can arise when rats do not have opportunities to engage in socially affiliative behaviour [23,24,25,26] and when social encounters are agonistic [5]. Accurate identification of affiliative and agonistic encounters, therefore, is likely to be paramount to ensure the welfare of rats in captivity (including pet and laboratory animals).

Wild Norway rats (*Rattus norvegicus*) live in colonies of upwards of 150 individuals [22], with these colonies consisting of subgroups made up of different combinations of individuals (e.g., pairs, harems, single mating pairs, etc.). It is unknown how these subgroups interact with the wider colony, however, or how stable they are [3]. However, information about rat social relationships has come from a number of behavioural studies [2,4,6,27,28,29,30,31,32]. In particular, work has been conducted on social status and dominance over many years [2,27,28,29,31,32]. Barnett (1958) described male rat social status in artificial colonies as having three categories: alphas (rats that were either equals or superior over other categories), betas (rats that were subordinate to the alphas, and adjusted to being under the alphas in the hierarchy), and omegas (rats that were subordinate to the alphas, although less adjusted than betas, bullied by the alphas and that did not generally survive for long) [2]. Dominance relationships have been shown to be stable over time [28,29,31,32].

Mauri, Bonelli, and Ozella, (2023) studied the social interactions of both male and female rats held in a post-laboratory animal care facility [4]. They described ‘ego networks’ of rats (i.e., specific connections and relationships), which were stable over time. This changed when new rats were introduced to the colony, but the stability of the colony was later restored. A greater number of positive interactions were observed than negative interactions, which may be a reason why these ego networks were generally stable. The authors reasoned that the space and enrichment provided for the colony were adequate and this was the reason for the higher number of positive interactions compared to negative interactions [4]. Furthermore, Proops et al. (2021) found that male rats in a laboratory setting formed differentiated social relationships, having individuals that they preferred to interact with, and individuals that they avoided when possible [6]. There has also been research on the structure of rat play behaviour, that has identified ‘pinning’ and ‘pouncing’ as being characteristic features [7,8]. It has been suggested that the body area that rats target when fighting allows play fighting to be distinguished from more serious fighting, with the nape area being the most common place for contact in the former, and flank and rump areas in the latter [7,8,9].

### 1.2. Pet Rats

Pet rat ownership in the UK is on the rise, with numbers climbing from 0.2 million rats owned in UK households in 2021 to 0.3 million in 2022 according to UK Pet Food [33]. Additionally, a 2022 BlueCross UK pet census found that 2% (1945.24) of respondents owned rats [34] and a 2021 Australian survey found that approximately 4% of Australian households owned small mammals, with 13% of these being rats and mice [35]. However, despite the growing number of households owning rats, little work has been done to study the behaviour and welfare of this population. In an attempt to rectify this, Neville et al., (2021; 2022; 2023) and Schneidewind et al., (2024) recently started to investigate this area, identifying how current pet rat keeping practices may influence rat welfare and developing guidelines for rat housing based on input from owners, vets, and animal welfare scientists [10,36,37,38].

Using a structured questionnaire study, Neville et al., (2022) reported that pet rat owners observed both climbing and communal sleeping daily, and allogrooming very frequently. In contrast, gnawing and boxing were less commonly observed behaviours, with biting (either another rat or a human) being infrequently observed [10]. This study also identified moderate correlations between several behaviour pairs: bruxing and boggling (both involving the masseter muscle), nesting and food hoarding, and behaviours associated with playfighting, such as boxing and chasing, boxing and pinning, and pinning and chasing [10]. Schneidewind et al., (2024) investigated social behaviours in more detail by asking owners to report the behaviours of a ‘focus rat’, finding that sleeping in the same house as a conspecific, huddling with a conspecific and eating peacefully with a conspecific were most commonly reported to be observed by owners multiple times per day. Furthermore, owners most commonly reported that their rat never plucked out fur, avoided contact with a conspecific, or bit a conspecific [37].

### 1.3. Owner Reports of Behavioural Interactions

In studies of the welfare of companion animals, owner reports of behaviour and behavioural interactions can be informative in a number of ways. For example, owner reports are a rich source of data that can provide insight into everyday behaviours that might not be caught in more targeted studies. Furthermore, studies based on owner reports can often be conducted on a large scale, allowing for high levels of statistical power in findings.

While limited in number, there are also studies that have addressed whether owner reports align with physiological indicators of welfare, as measured in experimental studies [39,40]. Increasing numbers of studies now utilise owner perceptions of their animals’ behaviour to provide an insight into the sorts of problems (e.g., welfare problems) that might be occurring within pet populations [10,15,36,37,41,42]. For example, Neville et al., (2022) investigated what information could be gathered from pet rat owners using an online survey of behavioural and husbandry-related information and whether this information could inform future studies on rat welfare [10]. Similarly, one of Schneidewind et al., (2024)’s aims involved providing insight into the husbandry, health, and behaviour of pet rats and the human–animal relationship [37]. However, it is worth noting that while useful for providing insight into issues, these studies do lack any kind of validation of owner reports and therefore need to be interpreted with some caution.

### 1.4. The Present Study

The present study employed interviews with pet rat owners, analysed using a reflective thematic analysis technique, to explore owner insights into their pets’ social behaviours, interactions and dynamics. Of particular interest were ‘positive’ (e.g., friendly, affiliative) and ‘negative’ (e.g., unfriendly, agonistic) social behaviours that were likely to relate significantly to the dynamics within the groups, and the welfare of the rats concerned. Many social behaviours (e.g., maternal care, play, and sexual behaviours) are thought to be associated with positive affective states in rats and the living conditions that allow or enable such states to arise [43]. However, there are also some social behaviours associated with negative affective states and living conditions that allow such states to arise. Examples include agonistic behaviours, and other behaviours indicative of social disruption (e.g., fearful and avoidant behaviours), and these are likely to be associated with poor welfare [3]. Certain living conditions and husbandry methods may prompt higher incidences of such behaviours (e.g., high stocking densities), and such behaviours themselves are also likely to impact negatively on welfare [44]. Pet rat owners were asked to identify such ‘positive’ and ‘negative’ behaviours and to describe the contexts in which they were seen. A reflective thematic analysis technique [45,46,47,48,49,50] was used to explore owner perceptions of these behaviours and the social dynamics within their rat groups more broadly.

## 2. Materials and Methods

### 2.1. Participant Recruitment

UK-based rat owners were recruited to take part in this study. An online post requesting participants for interviews was shared on six UK-based Facebook rat groups: ‘Rat Owners Group UK’, ‘Rat Owners South West UK’, ‘Rat People Bristol’, ‘Rat Lovers Wales’, ‘Scottish Rat Owners’, and ‘Rat Lovers UK’. The post asked people to share it further, allowing for snowball sampling. The eligibility criteria for taking part were that participants had to be 18 years or over and own at least two pet rats that currently lived together as a single group. Interested parties were provided with a participant information sheet and informed consent was obtained before interviews were arranged. Upon the completion of each interview, participants were sent a short Microsoft Forms survey (Appendix A) to collect demographic information. No incentives were provided.

### 2.2. Open-Ended Interviews

Open-ended interviews designed to explore rat owners’ perceptions of pet rat social behaviours and dynamics, were conducted between 24th August 2023 and 20th September 2023, and between 23rd January 2025 and 14th March 2025. Interviews were conducted online over Zoom (Zoom Communications (UK) PLC, London, UK), by a single researcher (CW), and recorded in video and audio. The interviews were semi-structured using a pre-arranged topic guide (below; see also Appendix B for full guide) and the researcher was free to further prompt participants to answer in more detail to ensure all of the three broad topics listed below were covered.

The interviews were structured around three broad topics: (1) owner perceptions of their rats’ relationships and social dynamics, (2) owner perceptions of positive or friendly social behaviours and interactions, and (3) owner perceptions of negative or unfriendly social behaviours and interactions. For ease of delivery, the topic guide was structured around four sections that fed into these three broad topics. The first part of the interview asked for basic information about the owners’ rats (how many they currently owned, their housing arrangements, sexes, neuter status, etc.) and details about their husbandry (cage type, cage contents, bedding/substrate used, feeding routine, etc.). The majority of data collected in this section pertained to a separate research question and is not presented here. The next part of the interview dealt with the relationships that the rats had and asked owners to detail the group dynamics of their rats (e.g., whether the owners felt that they got along, if there was an identifiable social hierarchy present, close friendships or conflicts within the group, etc.). The next part dealt with the reasons for these reports (what behaviours owners saw that led them to those conclusions), as well as asking owners for examples of positive interactions and negative interactions between the rats. The final part of the interview focussed on asking owners about how their rats’ relationships had evolved over time and about possible reasons for these changes.

Data saturation methods, specifically regarding the types of social relationships and behaviours reported, were undertaken to ensure interviews stopped when they no longer yielded new information relevant to the study aims. This was carried out as our focus was on the breadth of rat social relationships and behaviours. In line with the data saturation checklist proposed by Ahmed (2025) [51], a predefined saturation stopping point of no new types of social relationships or behaviours being mentioned after two consecutive interviews was set. Six to 12 individuals is generally believed to be a sufficient sample size for most qualitative studies [52]. For the present study, therefore, eight individuals were initially interviewed before the overall saturation of content was reviewed. Data saturation was not established at this point (following the checklist), however, so further interviews were conducted [51]. To implement this data saturation method, a saturation log was utilised where the researcher noted any mention of a previously unreported type of social relationship or behaviour. For the purposes of data saturation, ‘type’ was defined as a conceptually distinct social relationship or behaviour (e.g., sleeping together, allogrooming, or pinning). Additional instances or contexts of an already identified type (e.g., allogrooming in a different setting) were not counted as new types.

The interviews were recorded, transcribed, and checked for accuracy (by CW, the same researcher who conducted the interviews), before the original video/audio recordings were deleted. Once transcription was completed, the data were anonymised by replacing participant names with an anonymous identification number. Transcripts were imported into NVivo 12 Pro (QSR International UK Ltd., Warrington, UK) for reflective thematic analysis.

### 2.3. Reflective Thematic Analysis

Reflective thematic analysis was chosen because it allows for rich and nuanced exploration of participants’ experiences, which is particularly well-suited for studies that aim to explore complex, subjective experiences without being constrained by pre-existing frameworks or ideologies [53]. Furthermore, the flexibility that reflective thematic analysis allows for meant that the outcome of this study was not tied to one particular type of data or research question, but could adapt to further explore themes that came up in depth [45,46,47,48,49,50].

The analysis was conducted (by CW) using the step-by-step guide developed by Braun and Clarke (2006) [45] and further refined and outlined by Braun et al., (2022), Braun and Clarke (2019), Naeem at al., (2023), Byrne (2022), Braun and Clarke (2024), and Trainor and Bundon (2021) [46,47,48,49,50,54]. These steps can be summarised as: (1) transcribing the interview recordings in order to become familiar with the data; (2) going through the transcripts and noting down initial ‘codes’; codes are defined by Braun and Clarke (2006) as “a feature of the data (semantic content or latent) that appears interesting to the analyst and refer to ‘the most basic segment, or element, of the raw data or information that can be assessed in a meaningful way regarding the phenomenon’”; (3) placing codes into potential themes—broader categories that were based on similarity; (4) checking the codes placed into themes made sense; (5) refining the themes; (6) writing a report describing each main theme [45]. Even though these steps are laid out in a linear fashion, movement back and forth between the steps is an expected part of the reflective thematic analysis process [50].

All transcripts were coded using NVivo 12 Pro (QSR International UK Ltd., Warrington, UK) from Section 2 onwards (Section 1 just detailed demographic and background information), with many parts being coded multiple times (e.g., multiple codes could be mentioned within one sentence). An inductive ‘data-driven’ approach and semantic coding method was used with codes representing meaning as communicated by participants (i.e., the researcher did not attempt to attribute their own deeper meaning to what participants said) [49]. Codes were then grouped together, based on content, before being placed into potential themes. The themes were generated by the researcher’s interpretation of the data, rather than being pre-set, although they were partially informed by the questions outlined in the topic guide (see Appendix B). Themes were then refined by checking that the context and meaning of codes fit into the correct theme and also split further into sub-themes, where relevant.

In presenting the findings, participant excerpts were selected to illustrate each theme. Excerpts were chosen after coding and the theme refinement process was complete. Selection was guided by patterns in the data, with excerpts chosen to illustrate and deepen understanding of common responses for each theme. Excerpts with differing views were also purposefully chosen to reflect the breadth of perspectives and represent the full dataset. This is in line with recommendations for reporting quotes in qualitative research [55,56].

### 2.4. The Researcher

The researcher conducting the analysis (CW) is a white female in her 20s. CW is a PhD student focussing on pet rat social relationships. She has also owned pet rats for twelve years and is an active member of several online pet rat communities, allowing for valuable insights, and a rapport between the interviewer and interviewees, which may have allowed the interviewees to feel more comfortable and therefore be more forthcoming with information [57]. CW has experience with both good and bad pet rat social dynamics and rat introductions (both positive and negative) in her home life, which may have had an influence on her reflection of the data. CW’s personal interest in the topic and insider knowledge helped to inform the questions asked in the interviews and with participant recruitment, as well as to connect the resulting data with relevant research.

This was CW’s first reflective thematic analysis, and they were trained by two experienced colleagues. Reflective thematic analysis was chosen as the method for this study as it allowed CW to take an active role in the knowledge production and engage in a thoughtful and reflective way, taking her prior experience with pet rats into account [49].

### 2.5. Ethical Approval

Ethical approval for the study was granted by the University of Bristol Faculty of Health Sciences Faculty Research Ethics Committee (FREC; ethics number: 15556).

## 3. Results

### 3.1. Rat and Owner Demographic Information

A total of 23 participants were interviewed. This number was determined by using the data saturation checklist proposed by Ahmed (2025) [51]. The majority of participants were women (21) and fell into the age category of 25–34 years or 35–44 years (11 and 7, respectively). The most common total number of rats that a participant had owned in their lifetime was between 21 and 49 rats (7), with two participants having owned only two rats in their lifetime—with these rats being the subject of their interview. Demographic information about the owners is listed in Table 1.

The majority of owners in this study (n = 15) owned a single group of rats (a group was defined as rats permanently housed together in the same cage), six owners owned two groups and two owners owned three groups. The mean number of rats participants owned at the time of interviews was 6.8 ± 4.1 (mean ± SD) and ranged from two to 15 rats (median = 5). Table 2 details more information about the participants’ currently owned rats.

### 3.2. How Participants Responded to the Interview

The interviews were informal in nature and took on a conversational tone. The researcher asked open questions from the topic guide but also allowed participants to talk about what they wanted to, only giving further prompts if the original questions were not answered.

The length of time each interview took varied, with the shortest interview taking five minutes and the longest taking 41 minutes, depending on how much detail each participant went into and the number of rats they had to talk about. The median length of time interviews took was 20 minutes. Although much shorter than the others, as the interviewee that took five minutes did answer all of the questions, the responses to this interview were included.

### 3.3. Qualitative Themes

Given the largely open-ended nature of the interviews, discussions ranged widely, and the themes identified went beyond the original focus of the study on pet rat social behaviour and dynamics.

Sixteen themes emerged from the initial analysis (see Supplementary Materials File S1 for definitions and initial thematic map).

**Figure 1:**
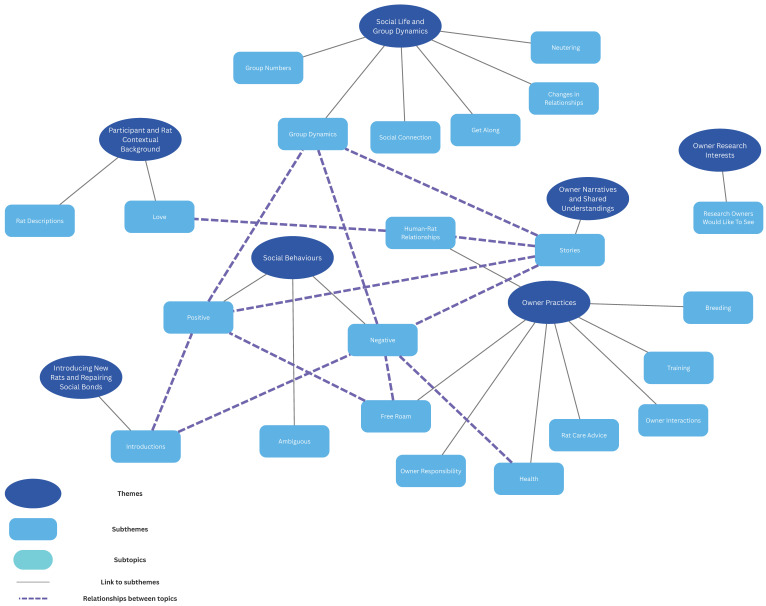
Final thematic map produced from open interviews. Dark blue ovals indicate a theme, blue rounded squares indicate subthemes, grey lines indicate a link to a subtheme (where the content of the subtheme related directly to the main theme), and broken purple lines show relationships between topics (where the content was less directly related but links could still be made).

Themes were then further refined by combining similar themes together (see Supplementary Materials File S2) and seven main themes were generated from the analysis: 1. Social Behaviours; 2. Social Life and Group Dynamics; 3. Introducing New Rats and Repairing Social Bonds; 4. Owner Practices; 5. Participant and Rat Contextual Background; 6. Owner Narratives and Shared Understandings; and 7. Owner Research Interests (Figure 1). The first three themes, Social Behaviours, Social Life and Group Dynamics, and Introducing New Rats and Repairing Social Bonds related directly to the initial focus of this study (rat social relationships and behaviours). The remaining themes, Owner Practices, Participant and Rat Contextual Background, Owner Narratives and Shared Understandings, and Owner Research Interests were also generated from the data analysis. Subthemes were formed where the content of the subtheme related directly to the main theme (e.g., positive, negative, and ambiguous behaviour subthemes for the theme of Social Behaviours) and relationships between topics (the broken purple lines in Figure 1) were identified where the content was less directly related but links could still be made (e.g., the subtheme of introductions could be linked to both positive and negative social behaviours as owners often talked about them in relation to these behaviours, but it was a distinct subtheme of Introducing New Rats and Repairing Social Bonds).

#### 3.3.1. Social Behaviours

This theme captures how owners interpret and narrate their rats’ behaviours. Owners discussed a number of behaviours that could be further broken down into the subthemes of ‘Positive’, ‘Negative’, and ‘Ambiguous’ behaviours. Within this structure, a behaviour was defined as ‘positive’ by the researcher if participants gave it as an example when asked about positive behaviours, (owners were asked ‘Can you give me any examples of positive/friendly social interactions within the group?’ and behaviours listed in their response were classified as positive). Similarly, negative behaviours were defined as such when given as examples of negative/unfriendly social interactions. Ambiguous behaviours were ones owners identified as having more than one meaning, depending on the context of the situation. Table 3 details the behaviours that owners perceived as positive, negative and ambiguous behaviours reported by participants in this study.

The only behaviour that was mentioned by all 23 participants was rats sleeping or cuddling up together. Owners used words such as “*cuddled*”, “*snuggle*”, and “*squash*” to describe how their rats slept together, with some emphasising that the rats chose to sleep together despite having the option not to.

*“…they cuddle up together, they sleep together. They don’t have to sleep together if they don’t want to—especially in the play pen, they’ve got loads of different places they could be. And occasionally they sleep separately, but most of the time they’ll squash into one setting.” [O3]*

*“They’re always cuddled together, like for sleeping and that. You never find... It’s very seldom you ever find one on their own.” [O14]*

Sleeping together was unanimously considered as a positive behaviour and it stood out as a clear indicator to owners of how well their rats were getting along.

The difference between rats simply sleeping together and rats sleeping together in a relaxed manner was also highlighted.

*“…sometimes they’ll be sleeping together, but they’re not 100% relaxed. Whereas sometimes when you look at them, they are. You can tell that they’re more comfortable being vulnerable around each other… I’m trying to articulate it. So, say you’ve got two rats sleeping next to each other, just it’s quite discreet. Whereas when they start to overlap, like have a leg hanging over each other, or like spooning each other, that kind of thing like when they’re a bit more, it’s not a technical term—mushed together.” [O1]*

This excerpt captures an important nuance in participants’ accounts: this owner emphasizes that behavioural context can be as significant—if not more so—than the behaviour itself in understanding what is going on. This highlights the value of situating rat behaviours within their relational and environmental contexts.

Grooming, specifically of companions, was another commonly mentioned behaviour interpreted positively by owners. Yet, while allogrooming was generally identified as a positive behaviour, owners did note that it could in some contexts be interpreted negatively.

*“Yeah, it’s the grooming that I kind of find interesting, so I know that’s a sign of them like bonding. But then it’s funny when it seems to kind of get a bit too much for someone and you hear them squeaking a little bit. But again, it’s such a like soft, low level squeaking. They’re not really trying to get away. They’re just kind of like lying on their side with their eyes closed… you hear these little peeps sort of coming out and someone’s like grooming their face or their head or their ears… it seems friendly.” [O3]*

*“…grooming each other, but not in a grabbing and pinning kind of way… a way where the groomee, or the one being groomed, is again relaxed and it’s not like a power play type thing.” [O1]*

*“She totally does have a bit of over grooming on her backside. She’s a bit threadbare there. So I think the others do take to grooming her quite a lot, but she doesn’t seem to complain. I never hear her make any sounds, and she’s been vocal in the past.” [O6]*

While owners do generally consider grooming to be a positive behaviour, it is again dependent on context. There is also a degree of ambiguity with this behaviour, with owners reporting that overgrooming may be fine as the rat being overgroomed does not complain.

Additionally, both sleeping in close proximity and grooming each other was seen by owners as a way of showing that other rats are accepted members of the group.

*“…including the new rat in the pile and grooming a new rat.” [O7]*

It appears that owners perceive both sleeping in close proximity and rats grooming each other as positive behaviours that are indicative of good relationships.

Playing was also commonly mentioned by owners as a positive behaviour. For the purposes of coding, mentions such as *“play fighting”* and *“wrestling”* (which solely occurred in response to the participants being prompted to describe positive behaviours) were therefore included in the sub-theme of positive behaviours.

*“I’ve noticed that quite a lot, there will be a bit of play fighting between them as well, which I consider a part—I know that when you’re a brand new rat owner, you’re kind of a bit like ‘oh’ about every kind of play fighting because you’re worried that they’re going to injure each other—but I think as you get more experience with rats, you just kind of accept that it’s part of the way that they interact with each other, and especially amongst the younger ones, there’s quite a lot of play fighting.” [O6]*

Chasing was frequently mentioned by owners in a positive light, and often described alongside playing, although chasing appears to prelude playing.

*“…the younger ones sort of chase and play a little bit, which is nice.” [O3]*

*“Sometimes they kind of prance around, like with each other, and start playing.” [O1]*

A notable aspect of playing behaviour appears to be the presence of other owner-defined positive behaviours.

*“…one of them will sort of pounce on the other and tip the other onto her back, and then they’ll kind of be a bit of sniffing, maybe a bit of grooming. After that it’s almost like a bit of a chase situation.” [O6]*

*“Hopping around the cage, definitely lots of hopping going on, running around the cage a lot, playing with them” [O8]*

Owners described play behaviour as light and reciprocal in nature. There was a stark contrast between how owners described these play behaviours and how owners described behaviours they perceived as more negative, particularly fighting.

Owners described fighting behaviour in a wide variety of ways, from “*tussles*” and “*argy bargy*” to more serious descriptions such as “*kick fight*” and “*aggressive*”. Notably, owners often described one particular rat within a group as the instigator of these fights.

*“He’d just pick fights with everyone.” [O2]*

*“…she’d actually get angry and sort of fighty…” [O3]*

Owners also described small fights that happened over items that were valuable to the rats, such as food.

*“…if you give them corn on the cob, everybody goes… it’s very predictable that there will be a fight at some point over food because it’s their favourite food…” [O4]*

There appears to be an accepted scale of fighting behaviours amongst owners.

*“So scaling up would be so that if they touch the other rat and they squeak, they’re not overly happy about that interaction… Yeah, sort of like squeaking. Tensing up. Kind of going more so on to their side. Not saying this like one hundred percent happens in my group, but they’ve been like odd little moments during intros. All that kind of thing, and sometimes if someone’s really worked up, they can get kind of like look a bit spiky with their hair fluffed up. Rat balls, where they just start fighting. There’s a bit of squeaking and it’s just a little tussle. More sort of like teeth baring that can be another bad sign. And almost like a sniffing kind of sound… And then obviously kind of like any scratches, bites.” [O1]*

The behaviour of chasing was also mentioned in a negative light. It was often mentioned alongside fighting, pinning, sidling, and kicking out with the back leg.

*“…chasing, fighting, consistently pinning when the other one’s really not liking it and the sidling, and that back leg kicking out.” [O7]*

Teeth bearing appears to serve a similar function to squeaking or tensing up: acting as a warning that the rat is not happy.

*“…literally as soon as one even comes up to him, his teeth are out.” [O4]*

Other notable negative behaviours included a rat being puffed up, humping, and boxing.

*“When [Rat 1] is trying to like hump say [Rat 2] for example, and then he gets all like puffed up and annoyed about it and starts like shoving his side into him. And then, like, they’ll sometimes start like boxing, where they both go on the hind legs and, like, do that, which is like a negative interaction.” [O16]*

*“Well, they… they like try and pin each other down or try and hump each other. And there’s only like once or twice they’ve pulled some fur out.” [O5]*

Biting was viewed particularly negatively by owners.

*“I have seen him biting actually as well… Biting on the back, but not hard enough to have caused you know, just like you know… Vicious bite.” [O7]*

*“We did have the biting, where they did have the, like I say, that big spat that was worrying me, where they did actually draw the blood from each other and they were baring teeth and fluffing up.” [O18]*

From these interviews, it appears that owners are able to identify a much higher number of negative behaviours than they are positive. One owner suggested a possible reason for this.

*“…it’s always the ways that it’s harder to notice the positives than the negatives cause the positives are small, quiet things and the negatives are things that make you go dashing over, going what on Earth’s going on in here.” [O7]*

This explanation seems to fit well with the data that have been collected: while owners are able to report some positive behaviours, they reported many more negative ones.

Owners also reported a number of behaviours that they were unsure about the valence of, or that the meaning differed due to context. These behaviours were therefore classified as ambiguous.

The first of these ambiguous behaviours is dominance grooming. Owners have clearly reported that they perceive grooming behaviour to be positive, even in potentially less positive contexts like overgrooming. However, owners have also suggested that more aggressive grooming is not necessarily positive or negative.

*“You can tell by the way they’re doing it. Like, what type… if it’s like a kind of more aggressive one, it’s more like a dominance thing. And then if it’s more like a gentle one, it’s like kind of more of a friendship.” [O16]*

*“So what I call it is vigorous grooming, so it kind of like where they groom each other. Like kind of like pin each other down and groom each other. And they’re sort of nibbling on them and they squeak like whoever’s being nibbled on, like, squeaks. And yeah, I call that vigorous grooming. So it’s kind of like probably is a bit of dominating…” [O19]*

There was a distinction between grooming types, with owners not classifying a certain type as positive or negative, but rather as a dominance behaviour—something that they have also reported to be a normal aspect of rat groups.

Owners also talked about behaviours they attributed to meaning extreme emotions and could therefore be considered either positive or negative behaviours depending on the wider context.

*“Something that I really like is when they are playing. They do wag their tail, which I kind of enjoy because it means… it can mean they’re like feeling extreme emotion. And when you can tell they’re fighting and they wag their tail, sometimes it’s like… When it’s going badly, and sometimes it’s when they’re really, really excited, rats are weird and sometimes it means both things, but that’s something that really stands out when they, like, wag their tail.” [O13]*

*“So at the start, it was when I first brought them home, it was quite kind of obviously of stress boggling, bruxing, trying to figure out all the new things in this cage that I’ve never been in before…. But now you can tell because they kind of… I don’t know if that was the right word but when a rat kind of like pancakes—makes himself flat and they’re all sleepy and their eyes are very sleepy. And then they start boggling, and that’s pretty cute. And they don’t move, you know.” [O10]*

This highlights the nuanced nature of behaviours that may signify extreme emotional states. A single behavioural display might indicate joy in one setting and distress in another, underscoring the importance of interpreting these behaviours within the framing of the wider context of the situation.

Owners also described the context around play behaviour helping to show the valence of those behaviours.

*“And you can kind of tell when it’s play fighting because it’s not getting too nasty. And also one of them isn’t kind of being like pinned or like one of them isn’t running away.” [O13]*

*“It can be fun when it’s playful, but if they’re puffed up at the same time, it’s like normally not… Cause you can see when they’re doing it, but like they’re sort of like letting each other win, you know, but when they’re not, no one’s like no one’s backing down. There’s like, tension there. I think that’s sort of a tell as well.” [O16]*

This owner used tension, or the lack of tension, as an indicator of whether rats are engaging in rough-and-tumble play or fighting.

#### 3.3.2. Social Life and Group Dynamics

This theme focuses on how rats related to one another within group settings. It includes how owners perceive and manage social hierarchies, group size, and the shifting dynamics between rats over time. This theme captures how rat groups are not static but socially negotiated, influenced by both rat behaviour and human intervention.

Participants in this study described multiple different perceived social dynamics in their rat groups. The most commonly reported dynamic was that of a clear leader—often described as an “*alpha*” rat. Some owners, however, reported that they could not determine a hierarchy with certainty. Others described configurations such as one rat attempting to dominate all others despite not being in charge, paired leaders, rotating dominance, and relationships where all rats were equal ranking.

Owners frequently described their rat groups as having a dominant or “*alpha*” rat.

*“[Rat’s name]’s definitely the boss.” [O14]*

*“We think we’ve got [Rat’s name] as the alpha…” [O18]*

*“[Rat’s name] is the, I believe the dominant female.” [O19]*

These responses were to be expected given the questioning about social hierarchies and whether or not there was a ‘boss rat’, but these terms were reported both quickly and consistently across interviewees.

Furthermore, owners commented that the most cohesive rat groups tended to be the ones where you could not tell who was in charge.

*“In general, I think that the best groups are the ones where you can’t really tell what the hierarchy is for sure. I think those are the ones that work the best. The worst groups that I’ve had is where you’ve had one rat who wants everyone to fall in line. That’s where things start to get a little bit more tricky.” [O1]*

This might suggest that where owners were able to describe a hierarchy in the group, the group itself was not particularly stable.

Despite this observation, owners frequently reported that their rats slept, cuddled, and generally spent time together when asked to describe the relationships within the rat group.

*“…these ones are always cuddling together.” [O10]*

*“Mostly they do get along like they’re always cuddling and sleeping together…” [O13]*

They also generally described their rats as getting along.

*“They’re all buddies really, to different extents I think.” [O1]*

This excerpt is particularly noteworthy as it reflects a nuanced perspective among rat owners: while there is a general belief that rats get along well, there is also an awareness that this is not universally true.

This message came up often throughout the interviews, with owners acknowledging that not all rats will get along but as social creatures, it is important for them to have rat company.

*“…it’s like any group, they’re always going to have fallings out.” [O11]*

*“…if you look at people, people don’t always get along, but actually we know how important it is for people to have other people. And obviously there’s a limit of like, I don’t want my animals to be scared or getting hurt by each other. But also like you, we know how important it is to have rats in a group so if there is going to be a bit of like fighting or a little bit of like negative behaviours every so often, that’s still definitely worth it compared to them living on their own.” [O21]*

Building on this, owners often talked about individual rats needing to have at least one ally within a group to make it tolerable for them.

*“Rats they are very social animals. I think it’s highly important that they’ve at least got one companion that they like.” [O8]*

*“I don’t like having just a pair of rats. I like them having options…” [O1]*

In fact, owners even commented on the numbers that they viewed as ideal for group dynamics.

*“…you should always have ideally sort of four or five because they just, they thrive in a group.” [O15]*

*“I’d say I like having at least a group of about five in terms of social interactions, I think a group of five gives rats options. It gives them different dynamics.” [O1]*

*“I’ve often had groups where you’ve had five rats, six rats or seven rats, and as the numbers go down, the dynamic changes within the group and all of a sudden it doesn’t work.” [O9]*

These owners considered that rats need options socially to thrive and that groups are better than pairs, which is not typically how rats are housed in laboratory settings, and suggests that further research might be valuable to determine if this is an area that could be improved within a laboratory context.

Owners also talked about their struggles in knowing what the right thing to do is when it comes to their rat group not getting along.

*“…from an owner’s perspective, I can imagine that if you had a group where there was a lot of, you know, I see on like rat care UK groups and things like that people getting really anxious over fighting and over the dynamics and, you know, should I neuter for the behaviours? Should I get the implant to, you know, make the group more cohesive? Or, people really struggling with the idea of maybe having to remove one or two or more of their group in order to kind of maintain the safety of some of the others. And I have had in some cases in past mischiefs that had... one of my girls, [Rat’s name], she was a great rat, but I don’t know whether she had just like, a hormonal shift, but she became an absolute nightmare at one point, and I ended up having the implant put in her. I would never have given her away or rehomed her, but I knew that I had to take action.” [O6]*

*“So if you feel as though you know, that some rats are being made unhappy or are unhappy as a result of actually being the one who has the hormonal aggression because I remember the first rat I ever had neutered for hormonal aggression, when I saw him afterwards, I was just like, oh my goodness, I realise now how stressed he was in himself. He was so tense and stressed all the time and actually it wasn’t just the impact on the others, it was actually on him.” [O7]*

Owners acknowledged that rat groups do not always get along. The solution to this is often neutering the aggressor in this scenario.

*“I mean, in the beginning it was like… [Rat’s name] was the dominant one and there was a lot of aggression… So that’s why he was neutered. But then after that, things have calmed down.” [O11]*

*“And luckily the neutering seems to have, like, really helped.” [O16]*

*“Then he became just overwhelmed with hormones. So he had to be neutered, and now he’s the most friendliest rat.” [O4]*

Neutering problematic rats appears to have the desired effect of calming the group dynamics back down.

Owners also acknowledged that the dynamics within rat group change over time, and especially as rats are lost and new rats are added.

*“I have quite a big rolling group that they kind of adapt to, you know, the losses of certain ones. I do notice you know, whenever I lose some... like one of them who is quite dominant in the group or you know, more elderly that there will be a change in dynamic for a week or so and then you know they start to reestablish that dominance. And yeah, and I suppose coming from when the babies are there and we’re doing intros and stuff, the babies will be absolutely going haywire, and the older ones will kind of be like, calm down, you know? So that kind of keels and mellows as they grow up.” [O12]*

The idea of a rolling group came up frequently and so changes in rat group dynamics are likely to be common. This sets the scene for the kind of social dynamics that pet rats experience, which will be explored further in subsequent sections.

#### 3.3.3. Introducing New Rats and Repairing Social Bonds

This theme focuses on the process and challenges of adding new rats to an existing group or restoring harmony after conflict.

Where owners detailed how they performed these introductions, they all described doing the ‘Carrier Method’—a form of introduction not previously mentioned in the scientific literature.

*“So basically we do the carrier method… where you put them in a really small area, basically. I think we started off by just bonding a couple of them at a time in the different groups. Sort of like one or two at time, the more friendly, relaxed ones kind of thing… And then we sort of like added them when things were going well. So there was like loads of treats and stuff. And being in the small space… So yes, so we put lots of treats in like a small kind of carrier, like a small pet carrier sort of thing. So it means they couldn’t like run away from each other and you just kind of like watch them like a hawk to make sure there’s no like signs of aggression sort of thing. So we did that and eventually got them all together, within like kind of a few hours and then we just kind of stayed with them for the first few days.” [O17]*

*“Alright, you start off with a small carrier. I’ve got varying sizes so I start off with the smallest one which is, depending on what size of rats you’ve got. The boys went into a medium size because they’re big ones now. The girls, because they’re tiny little girls, they went into the smallest hand carrier one and they stayed in there for a while. And I basically just put them on the bed next to me and I just lay there. All they’ve got is them and some substrate under them and it’s basically just silence and them. And it’s just I’m watching them to see what they do, if there’s any arguments. If there is, then I can just lift them out straight away. I’ll wait to see what happens. They settled themselves down after lots of sniffing, scratching around each other, grooming each other, more sniffing, more licking and eventually they just settled. And what you want is to watch them sleep together, not as a pile of the old rats and the new rats on either side. You want them to be a cuddle pile together. And they did that. Then you move to a bigger cage and you want the same with more space. So they’ve got more space to move, but you want them to do the same. And so they did that. So then I put them in the cage that they’re in now, but with the hammock and a litter tray. And then for them to see if they would use the hammock together. And they did. And they all went into the hammock, and they slept in the hammock together. They just went beautifully. I skipped other steps because they just went in so beautifully.” [O18]*

*“I do the carrier method for introductions so they start off in a small tank, then up to hamster cage up to an Alaska and then into their bigger cage and I start off with just their substrate and their food in the tank. And then when they get up to hamster cage, I’ll give them a hammock once they’ve kind of settled in and once they’re snuggling together. So until they’re snuggling together, they don’t get to move up a stage. I have done introductions in one day when we have started at nine in the morning and I’ve put, like 6 rats in a hamster cage. And then by the end of the day, then they’re full cage and they’re starting to get their toys and their hammocks and everything back.” [O14]*

The carrier method appears to involve introducing unfamiliar rats in a small neutral space and making sure they are getting along well before increasing the size of the neutral space. If rats continue to get along, they will then be placed in the cage intended to be their home and enrichment items are added over time, making sure that the rats continue to get along during this period. It was also clear from what the owners were saying that they are very involved in this process and watch over the rats to make sure introductions go well.

Owners introduced new rats to their existing group of rats to have a rolling group.

*“[Rat’s name] is now the main boss, but it will be interesting when we introduce the two new ones to see what will happen with the dynamics there…” [O11]*

Additionally, owners also went through the process of reintroducing rats to re-establish social bonds when they were not getting along well.

*“They had a spat recently. I think they were having a bit of a who’s boss and working out if they were changing like who’s the alpha in the group. So we did a bit of rebonding and they actually spent a good old time doing the carrier method again and they spent some time back in the carrier and getting back into that and it seems to have settled it down. And they’re back, all grooming each other. And they’re not having the spats that they were. And I think they’ve sorted out whatever was going on between them.” [O18]*

Despite the positive undertones conveyed when owners discussed rat introductions as a whole, many also acknowledged that it often was not without its issues.

*“But at first when I first integrated them, they… what happened was they had those like little scraps or whatever. But then I saw them not sleeping together during the day. And that’s when I took a load of the stuff out that I’d put into the cage. I felt that they were being territorial. And they were because once I took like the things out that meant... Because they were sleeping in separate areas, I took one of those areas out, then they slept together. So it’s kind of like forcing them… But then over time I’ve got everything in there now and they still sleep together like they choose to do that.” [O19]*

*“So it took a while to get them properly bonded and they did keep having scraps for, like, months actually.” [O22]*

The level to which scraps were tolerated varied, although one owner shared the advice that they had been given for introducing rats.

*“It’s the thing in the rat community that we say ‘if it’s no blood, no foul’, so it’s mainly if you don’t see any bites or anything and it’s just them being a bit bullish and just going no, no, no.” [O18]*

#### 3.3.4. Owner Practices

This theme explores the human–rat relationship as well as the active role of owners in shaping their rats’ daily lives. It draws together personal stories and moments that illustrate the emotional bond experienced by those who keep rats.

Owners frequently talked about their rats being friendly and having positive interactions with them.

*“So like [Rat 1] and [Rat 2] are extremely friendly like they come to the cage door.” [O14]*

*“He’s very, very cuddly with humans…” [O13]*

Furthermore, owners described the bonds that they have formed with their pet rats.

*“Oh yeah, they’re literally my little babies. Like ask anyone, they know like they mean the absolute world to me. I can’t imagine my life without them now. I’ve owned rats for four, five years now. And I just can’t imagine not having them in the house.” [O8]*

*“She was like my heart rat... So not my favourite or anything, but just we had that bond that was like next level.” [O17]*

*“He’s probably I’d say my favourite. I shouldn’t have favourites but he is my heart rat.” [O18]*

The term ‘heart rat’ conveys the genuineness of the bond that owners have spoken about.

This bond that owners spoke about appears to be reciprocated by the rats in question.

*“…happily accept strokes, and boggles…” [O10]*

*“I have never had a rat that loves me like he does. And he just loves so much. He will just kiss, kiss, kiss, kiss, kiss, kiss, kiss, kiss, kiss with all his heart, he just loves to cuddle and kiss… He just wants to kiss and he doesn’t do it really with anybody else. He just does it with me.” [O18]*

The love that owners feel towards their rats is so powerful. Likewise, it appears that many rats happily reciprocate these feelings.

Owners also described the interactions that they shared with their rats.

*“Oh yeah, they have a lot of free roam time on the bed. We have like… kind of make it into like a little assault type of course for them. So I have multiple little beds and toys out…. they really like the kind of cat teaser toys, with feathers on the end, they chased it around the bed. When they’re more… They’re still quite young when they’re more kind of old and cuddly, you know, like able to go elsewhere. I mean my old rats actually, used to have them everywhere.” [O10]*

*“And then when they come out, they get free roam of the lounge, they can go anywhere in the lounge, they can go all over the sofas all over because it’s hard flooring. They go anywhere and they climb onto the sofas, onto us.” [O18]*

*“I made them little like Christmas presents, just like a tiny little cardboard box filled with treats and then like tied up.” [O16]*

There is a real depth to the bond owners feel for their rats that has been highlighted in the above excerpts.

#### 3.3.5. Participant and Rat Contextual Background

This theme focuses on how owners describe their rats and their relationships with them, setting the scene for understanding the relationships within and between rat groups.

Owners often described their rats as little characters.

*“[Rat’s name] is the goofy one.” [O1]*

*“the queen of the cage” [O6]*

These excerpts illustrate how owners attribute personality traits to their pet rats. It is worth considering how such characterisations might shape participants’ accounts of social connection and companionship. Given that this study centres on owner perspectives, this interpretive framing is a key analytical consideration.

Furthermore, such expressions of character were often said with a loving undertone.

*“…they are all proper little characters and people don’t think that they are, but they really are little characters…” [O18]*

There was a feeling conveyed with this excerpt that rats are often misunderstood, but not by their owners.

In fact, owners often expressed their love for their rats throughout the interviews.

*“I love them.” [O1]*

It is important to note this lens of affection that owners so frequently conveyed when viewing the final dataset, as these feelings are likely to have coloured their responses.

#### 3.3.6. Owner Narratives and Shared Understandings

This theme draws together personal stories and moments that illustrate and provide more context for many of the reports throughout this paper. Many of these stories are quite lengthy so have been described in Appendix C instead of the main text.

*“I originally had the litter tray in the corner, sort of balanced almost on the substrate, obviously clicked into the cage corner. I’ve now had to up it, so it’s probably about twenty centimetres off the floor because what happened before when it was close to the substrate is one of the smaller males dug so he could get under it. But then, because [Rat 1] was that bit bigger, he couldn’t get into the hole that they had dug. So they would just be like shouting at him from the hole entrance, but obviously then they couldn’t really get out unless he dug himself out. So he’d be like panicking under the toilet, which was quite irritating and then more recently I put in a small box because to give [Rat 2] a bit of a space when he was feeling unwell and they went in there to hide, and [Rat 1] was just sort of guarding the door and just sort of screaming at him from the entrance so they couldn’t get out.” [O21]*

This excerpt helps to add some context by providing a scenario around some negative rat behaviours within the group.

An owner also talked about seeing grief in one of their rats.

*“About a year ago… basically them two were two sisters… That was [Rat 1] and [Rat 2]. And when [Rat 2] had been put to sleep, we had got home and we had sort of showed her sisters like her body sort of thing, just so they could understand. And it’s like, okay, maybe it’s not like a positive experience, but basically [Rat 1] kind of started squealing sort of thing and it’s just like because she was, like, heartbroken that her sister, who she’d been protected and looked after by and stuff, like her sister had gone. So it’s like... It’s not happy, but it’s like in terms of like loving.” [O17]*

This is a particularly powerful excerpt and illustrates how important rat social bonds are.

#### 3.3.7. Owner Research Interests

This theme captures what participants want in terms of further research on pet rats. It positions rat owners not just as caregivers, but as engaged participants in knowledge-making—expressing desires for more information and scientific validation.

Rat owners expressed that they were glad that research was being conducted on pet rats.

*“It’s nice that someone’s doing something with rats.” [O9]*

*“We definitely need more out there about it.” [O7]*

Owners also acknowledged that current rat care advice varies depending on where you look for it, and that there does not appear to be any one established place for advice.

*“It’s a shame that like with rats there doesn’t seem to be as much, you know with dogs, you know, there’s so much information out there that you can use and you can say, ohh, gonna you know go to a dog trainer and you know if your dog’s not getting on with another dog in the house, there’s like all these advice and things you can do whereas with rats I feel like, yeah, there’s advice on the internet, but it’s just other people on Facebook or something like that, which you never know if it’s... There seems to be a lot of conflicting advice. So it’s quite hard to know… And some of the stuff here online that rat owners suggest seems to sort of be at odds with that, but it’s almost like there’s no alternate voice saying something like that. So on these Facebook groups, apparently, you know, they all recommend a certain way of introducing rats to each other, and no one recommends anything else. And if someone does recommend something else, everyone says no that’s really dangerous, it’s a really bad thing to do. So yeah, it’s almost like you’re in conflict what the best way to do things is and there’s very little, very you know, kind of… Like if I had a dog, I’d know where to go to get like, you know, good information about dog training, I’d know what… who to trust. Whereas I think with rats, there isn’t that much established stuff out there.” [O3]*

While considerable research has focused on laboratory rats, there remains a notable gap in understanding pet rats, which are comparatively underrepresented. Furthermore, it is unclear whether research focused on understanding laboratory rat behaviour and improving their welfare has been shared with the pet rat community. It could therefore be beneficial to promote knowledge exchange between the pet rat community and laboratory animal specialists.

One owner in particular, expressed that they would like to see some research done into rat introductions.

*“I’d love to see, you know, I’d really like to see some research on you know what the effects on like cortisol or something like that of the rats that are being introduced to the new rats.” [O3]*

#### 3.3.8. Researcher’s Reflections

An integral part of reflective thematic analysis is the researcher’s reflections and acknowledgement of how their own positionality and interpretation shapes the generation of themes from the data [46,54,58,59]. For transparency, a summary of CW’s reflection diary is included in Supplementary Materials File S3.

## 4. Discussion

Understanding the social relationships and behaviours of pet rats can provide insight into this often underrepresented group of animals and may also provide information that could be applied to improve the welfare of both pet and laboratory rats. This study used qualitative methods to explore how owners perceive and manage social relationships within pet rat groups and investigated which behaviours they identified as social, including the context and valence of these behaviours. Owners were highly consistent in how they identified a number of social dynamics and behaviours, as well as in how they described their management of the social relationships within their rat groups. Owners consistently reported rats sleeping in close proximity and playing as positive behaviours. Allogrooming was also generally reported to be a positive behaviour, although there were contexts where it was reported as neutral (dominant grooming) or ambiguous (overgrooming). Fighting was consistently reported as a negative behaviour, with tensing up, moving sideways, puffing up fur, teeth baring, and a sniffing sound all being reported as warning signs rats can exhibit before a proper fight breaks out. Some owners also identified behaviours that convey extreme emotions (both positive and negative), such as boggling and tail wagging. Multiple different perceived social dynamics were described, of which, the most common was that a group had a clear leader, or ‘alpha’ rat. Owners generally believed their rats to get along with each other, but this was also reported to not be universally true—raising important questions about the implications of housing rats together that do not get along. This study includes the first academic report of pet rat introductions and introduces the ‘Carrier Method’. It was clear throughout that owners have formed strong bonds with their rats, which may be linked to an anthropomorphic framing of these pets. Furthermore, multiple owners reported having a “heart rat”—an expression not previously reported in the scientific literature and is used to convey the special and emotional bond that many owners described. Finally, rat owners were enthusiastic about research being conducted on the pet rat population, a potential avenue for future symbiotic relationships between researchers and the pet rat community. While our findings lack the quantitative nature of many of the studies discussed below, its exploratory nature offers complementary insights into owner perceptions of their rats’ relationships and behaviours.

The majority of owners in this study were female and fell into either the age category 25–34 years or 35–44 years. This is largely consistent with response demographics in previous studies of rat owners [37]. The mean and median numbers of rats participants owned at the time of the interviews (mean = 6.8 ± 4.1 SD, median = 5) was also similar to the mean and median numbers reported by Schneidewind et al., (2024) (mean = 7.0 ± 9.4 SD, median = 5) [37].

### 4.1. Social Behaviours

Sleeping in close proximity was generally perceived positively by owners. This is likely to be a common behaviour across pet rats: a recent questionnaire of pet rat owners found that communal sleeping, defined as rats sleeping in close proximity to each other, was reported to be observed daily by most owners [10]. This is also consistent with work on social behaviour in rats in the wild and in laboratory settings where “*huddling*” is interpreted positively [3].

Rats grooming each other, also known as allogrooming, was also generally perceived by owners as a positive behaviour. Allogrooming is a frequently observed behaviour in pet rats [10], and can be interpreted positively when it occurs in rats in the wild and in a laboratory setting [60]. However, owners also noted that allogrooming could be interpreted negatively and that vocalisations could be used as a distinguishing feature of more negative allogrooming. This is consistent with previous observations of wild rats that defined aggressive allogrooming as “often accompanied by squeaks” [3].

Allogrooming has been shown to be a reciprocal behaviour in outbred offspring of wild rats within a laboratory context, with both dominant and subordinate rats grooming each other (although dominant rats have been found to be groomed more frequently than subordinate ones) [60]. Thus, allogrooming may provide insight into the social structure of rats as well as cohesiveness of a social group, and may accordingly be a valuable behaviour to investigate from a welfare perspective.

Owners also made a distinction between grooming types in this study. They reported allogrooming (which was identified as positive), overgrooming (which was more ambiguous with some owners reporting that it could be positive and others negative), and dominant grooming (which was not identified as positive or negative, but rather as a normal aspect of rat groups). The exact cause of barbering, a type of overgrooming that has been studied widely in laboratory rodents (particularly in mice), in unknown, although there are several theories as to its cause including: dominance (disputed by several studies), genetics, social learning, boredom due to inadequate housing, and dietary deficiencies [61,62,63,64,65]. Van den Broek et al., (1993) suggested that barbering in mouse pairs is a mutual behaviour: where both parties receive some whisker trimming. They also suggested that whisker trimming could be a sign of inadequate housing, due to the reduction in (but not elimination of) the behaviour when a wire screen was introduced to the housing, allowing mice to separate themselves if they wished [66]. It is worth noting that this behaviour is not observed in wild-type mice living in their natural habitat, but rather in confined individuals and as such, is likely to indicate a poor state of welfare. Dufour and Garner, (2010) detail the mechanisms behind, and theories of, barbering, concluding that barbering is not a healthy social behaviour [65]. This suggests that barbering in rats, and perhaps overgrooming, is a sign of a poor welfare state—and perhaps even of a negative social relationship. In the present study, some participants did not describe overgrooming in this way, instead choosing to believe it was not negative due to the rat being overgroomed not appearing (to the owner) to exhibit signs of discomfort. It is possible that anthropomorphism has influenced owner perception of this overgrooming behaviour and the wider implications on the welfare of the rat in question [67]. Schneidewind et al., (2024) found that the majority of owners (91.5%) reported that their rats never plucked out the fur of a conspecific [37]. While this number suggests that overgrooming is not generally a problem in pet rats, as with the present study, participation bias is likely in the Schneidewind et al., (2024) study [37], and therefore, this view may not be representative of the wider pet rat population. More research into the different grooming types identified in this study could be a useful way forward in understanding the social implications of allogrooming.

Play fighting was noted by owners as a positive behaviour, and was labelled by them using terms such as wrestling, hopping, pouncing, and chasing. More generally, it was described as light and reciprocal. More agonistic fighting, on the other hand, was identified as being associated with kicking, teeth-baring, and pinning, and rat owners used visual signs of tension (e.g., a rat’s fur being puffed up) as a general indicator of fighting in this scenario. Interestingly, these reports differ somewhat from behavioural studies’ differentiations of play and agonistic fighting. Panksepp, (1981), for example, characterized play in rats through pinning behaviour, with chasing, pouncing, and rolling over each other also being a part of the play repertoire [68]. Play fighting has also been distinguished from agonistic fighting through its predominant focus on the nape [69]. Essentially, it seems that owners were more ready to report general, heuristic markers of playful versus agonistic behaviours, unlike traditional ethologists who have tended to rely solely on individual behavioural markers. It would be valuable to explore these differences further in future studies.

Almost all of the behavioural signs associated with fighting were identified as negative by owners. It appears that rat owners perceive there to be a scale of negative fighting behaviours: squeaking, becoming tense and moving sideways were perceived as warning signs of a negative interaction. Additionally, fur puffing up, teeth baring and a sniffing sound were also perceived indicators of a negative interaction. Scott and Fredericson, (1951) described rat fighting behaviour in detail: hair-fluffing and defence postures (such as rearing up) were commonly observed before the onset of actual fighting [70]. Owners in the current study described rats having puffed up or “*spiky*” fur before full on fighting breaks out. Owners also described a number of defensive behaviours such as sidling (approaching another rat sideways or backwards), pushing each other, rearing up, and boxing. Similarly, Scott, (1966) outlined a number of patterns of agonistic behaviours in rats. Many of these behaviours, such as prancing, approaching sideways, rearing and pushing (together as boxing), chasing, squealing, rearing up as a defensive posture, hair fluffing, and running away, were also identified in the current study [71], with many of the behaviours listed by Scott, (1966) also perceived by owners in this study to be negative.

Timmermans (1978) categorized agonistic behaviour between rats as offensive (where a rat attacks another, e.g., to gain or defend a territory), defensive (where a rat defends themself from another rat), and object competitive (where a rat performs agonistic behaviours to gain or keep items valuable to it, e.g., food or nesting materials) [72]. Offensive behaviours include a sideways attack (referred to as sidling in this study), fighting, chasing, biting, aggressive grooming, boxing, and pulling the fur of another rat. Defensive behaviours include freezing, crouching low to the ground, and running away. Many behaviours that are classified as offensive, such as fighting and biting, can also be classified as defensive where the rat in question is performing the behaviour in response to an attack [72]. Timmermans (1978) categorized object competitive antagonistic behaviour separately from the first two; with offensive and defensive behaviours the rat performing these behaviours directs its attention towards the other rat, but in the case of object competitive behaviours, the rat is focused on the object it is trying to secure [72]. Owners in the present study mentioned both offensive and defensive agonistic behaviours, talking about rats attacking another rat and how that rat defended itself (e.g., teeth baring or fighting back). Additionally, owners also discussed smaller fights over valuable items, such as corn on the cob, that could be classified as object-competitive agonistic behaviours.

Blanchard and Blanchard (1977) analysed the attack and defence behaviours of dominant (or alpha) laboratory rats when unfamiliar rats were introduced to the colony. They described preattack behaviours, such as piloerection and tooth chattering, that escalated into an active attack, with behaviours such as chasing, offensive sideways lateral attack (referred to as sidling in this study), and boxing [73]. The behaviours, and their escalation, are similar to owner reports in this study, and the scale provided by one owner describing the escalation of negative behaviours between rats.

Many of the behaviours perceived as negative by owners in this study (e.g., boxing and biting a conspecific) were reported infrequently in both the Neville (2022) and Schneidewind et al., (2024) studies [10,37]. A possible reason for this could be that both studies asked for general reports of behaviours (how frequently behaviours were observed and the frequency of behaviours observed in the last month respectively), whereas the present study allowed for owners to recall examples from time periods that were not specific to the present (e.g., back when introductions were taking place) so we may have captured more perceived negative behaviours due to this.

Notably, there are some inconsistencies between Schweinfurth (2020)’s proposed ethogram of rat social behaviours, split into behaviours that are ‘socio-positive’ (e.g., allogrooming, play fighting, and sharing food) and ‘socio-negative’ (e.g., aggressive grooming, chasing, and fighting) [3] and the behaviours reported here by pet rat owners. For example, it is possible that bruxing (which has been referred to as teeth chattering [37]) can occur in both positive and negative contexts [10,37], but it is solely given as a ‘socio-negative’ behaviour by Schweinfurth (2020) [3]. The behaviour of boggling that co-occurs with bruxing in rats [10] was reported by owners in this study. It was suggested as a behaviour that conveys extreme emotions, both positive and negative. As bruxing behaviour has been suggested to be either positive or negative depending on the context, and bruxing and boggling are closely related, it is possible that this is also the case with boggling. Schneidewind et al., (2024) includes tooth chattering both performed by a rat (and also referred to as bruxing) and as directed at a conspecific, with the former being reported more frequently than the latter [37]. This suggests that owners in other pet rat studies have also made a similar distinction. Further research is required to determine if this is the case, but it is important to note that context (that is likely to vary between laboratory and pet settings) may play a role in the valence of this behaviour.

It is also worth noting that housing configuration (e.g., pair housing vs. group housing) and sex may influence social behaviours. While it was not possible to investigate in the current study, this would be a useful next step for further research.

### 4.2. Social Life and Group Dynamics

The social relationships and group dynamics of rats have been explored previously in observational research, but not in a pet context. Previous work studying a female–male rat group held in a post-laboratory animal care facility suggested that rats form differentiated social relationships, preferring the company of some individuals over others [4]. Similarly, male rats in a laboratory setting have been shown to form similar preference-based relationships [6]. Furthermore, a study in a post-laboratory animal care facility observed a greater number of positive behaviours compared to negative ones, which may have been the reason why the ‘ego-networks’ they detected (i.e., specific connections and relationships) of rats were generally positive [4].

Participants in this study offered a broadly similar view. Furthermore, owners reported that they used signs of rats sleeping in close proximity to one another and grooming each other as indicators that the group was getting along well. This is consistent with previous literature suggesting that allogrooming may play a role within the social structure of rat groups [60]. It is therefore possible that allogrooming and sleeping in close proximity could be used as indicators of a cohesive social rat group. It would be interesting to investigate the relationships between these behaviours and behavioural and physiological markers of welfare.

With regard to social dynamics, Barnett (1958) described three social statuses in artificial colonies of male rats: alphas, betas and omegas [2]. Such classifications appear to have popular appeal: in the present study, owners readily identified who they believed to be the alpha rats in their groups. Interestingly, however, owners also identified that some of the best hierarchies were the ones where there did not appear to be a clear ‘alpha’ rat, suggesting that groups where an ‘alpha’ rat was clear (e.g., dominating behaviour was clearly observed from one rat) may not be as cohesive as previously believed. Further research will be needed to establish what behaviours might be best used as a proxy for cohesive and non-cohesive rat groups.

Puentes-Escamilla et al., (2025) tested the dominance hierarchy of both pairs and groups of outbred wild-type strain rats and found that both pairs and groups had stable dominance hierarchies over time, but that this stability was stronger in pairs compared to groups. The authors gave a possible suggestion for this: in groups, there is the opportunity for bystander rats to intervene [31]. Many owners in this study talked about having an ideal number of rats in a group (four to seven) and that having more rats in a group helped to give rats more options socially and dilute problems between rats. This appears to be in agreement with the findings of Puentes-Escamilla et al., (2025) [31]. Further research investigating how group numbers may influence the stability of group hierarchies, and the subsequent cohesion of the groups, would be useful to understand rat group dynamics.

While there was a general belief that rats got along well, many owners acknowledged that this was not universally true. Despite this, owners reported that it was important for rats to be housed together as they are social animals. This raises some important welfare questions, such as whether there are scenarios in which being housed singly would be more beneficial for the rat in question, and what the welfare implications are of housing rats that do not get along together.

### 4.3. Introducing New Rats and Repairing Social Bonds

Owners reported that they used introductions for two distinct purposes: to introduce groups of unfamiliar rats to each other and as a way to repair social bonds where they identified there were problems. They talked about rats having *“scraps”* when initially introduced, before the relationships settled down. In a study of a post-laboratory rat colony, Mauri et al., (2023) found a high level of social stability during the first study period, but when new individuals were added to the group, this caused a period of instability while social relationships were redetermined, before the social stability was restored [4]. This period of instability before stability was then restored may be similar to what rat owners are reporting when they report that there were initially scraps before the group became properly bonded.

This study includes the first academic report of introductions within the pet rat population. Participants described the ‘Carrier Method’ where unfamiliar rats are placed inside a small, contained but otherwise empty space (e.g., a pet carrier). These rats are supervised and if they settle together in the carrier, rather than fight or try to stay away from each other, they are given a larger but still contained space. These spaces increase provided the rats continue to get along, until the rats are inside the cage intended to be their home. Owners also reported that they introduce enrichment items slowly into the cage, often only giving the rats one sleeping spot initially to encourage them to sleep together.

While not reported in the scientific literature, the carrier method appears to be a widely known introduction method in the pet rat world. It is, however, not the only method, with popular rat care websites (e.g., http://www.isamurats.co.uk/introducing-rats/, accessed on 27 August 2025) describing other methods, such as the ‘Neutral Space Method’ (a more gradual introduction style conducted in a neutral space) and the ‘Heavy Supervision Method’ (similar to the neutral space method, but with intervention at any signs of agonistic behaviours). The carrier method appears similar to the stress bonding technique used in some rabbit introductions to bond rabbits by putting them in stressful situations (e.g., a confined space where they are forced to be in close contact) so that they rely on each other for comfort [74]. Social buffering, where the presence of a conspecific suppresses behavioural and physiological responses to a fear conditioned stimulus, has been documented in rats [75]. While this is not the same as stress bonding, it may explain the rationale behind it: stressed animals may seek closeness, which may lead to a calming effect and thus, the animals bonding.

The carrier method for rats is also notably different from the recommended method for introducing pet rabbits to one another (a very gradual and heavily supervised process [76,77]). The recommended method for introducing pet rabbits appears similar to the neutral space and heavy supervision methods for introducing pet rats. This prompts consideration of whether the carrier method (a method analogous to stress bonding) is the most appropriate method from a welfare perspective, particularly given the availability of alternative approaches.

Although not explored in this study, it is likely that individual differences, such as rat age, sex, and previous experiences may influence the success of introductions. Furthermore, participants in the present study did report introductions that did not work, suggesting neutering as a way forward before restarting the introductions again. This suggests that neuter status may also be an important consideration when introducing pet rats.

Rooney et al., (2014) proposed that further research is required to identify optimal methods of introductions in rabbits [78], and we concur that this would also be valuable for the pet rat population.

### 4.4. Owner Practices

As in Dickson et al., (2019), participants in this study reported having strong bonds with their rats, with some owners even describing their rats as their *“babies”* [79]. As the majority of pet owners consider their pets to be a member of the family [80], comments like these are unsurprising. While such language does not represent the authors’ own position, participants describing their rats in this way indicates an anthropomorphic framing that may influence owner perceptions of welfare needs and decisions related to appropriate care management of their rats.

A systematic review of pet-parenting grouped articles on this topic into three categories: terminology around pet-parenting, factors affecting the pet-parenting role, and pet parenting styles [81]. Additionally, different pet-parenting styles have been shown to predict social and problem solving behaviour in dogs [82] so it would make sense that pet-parenting styles may have an impact on the behaviours of other species as well.

While the current article does not directly assess pet-parenting in rats, many of the participants in this study used terminology (e.g., referring to their rats as their *“babies”*) that would indicate they identify as taking on a parental role in their human–rat relationship [81,83]. This framing could have mixed implications. Strong owner–pet bonds have the potential to enhance the animal’s welfare (e.g., through increasing the owner’s time and financial contributions towards their pet’s care). However, owners with very strong bonds may have difficulty, or show reluctance, accepting euthanasia as the best option when their pet is suffering [84]. Furthermore, anthropomorphism may cause the misinterpretation of behavioural signs of distress or inadvertently deprive animals of opportunities to express their natural behaviours. In the present study, this perception among some participants may have shaped their reported attitudes and practices, introducing potential bias into self-reported data. Awareness of such influences is important for interpreting the findings and for designing future research, where pet-parenting perspectives could represent both a potential confounding factor and a useful avenue for fostering participant engagement.

Multiple rat owners reported having a ‘heart rat’, a term not previously reported in the scientific literature. These owners discussed how these rats were rats with whom they shared an exceptionally special bond. In Neville et al., (2021), nearly one third of rat owners reported that they liked the relationship they felt their rats had with them [36]. Schneidewind (2024) found that the mean attachment to pet rats using the Comfort from Companion Animal Scale was skewed towards more attached (mean of 37.8 ± 5.3, median of 38 and a range of 15 to 44 with possible scores ranging from 11 to 44) [37]. Despite only a handful of studies investigating the human–rat bond thus far, it would appear that rat owners have a strong connection to their pets. This could have a positive influence on pet rat welfare with owners putting more time, effort and money into providing species-appropriate care for their rats or a more negative influence if owners are too attached to their rats to make necessary decisions to promote their welfare (e.g., humane euthanasia or rehoming where they no longer have the resources to provide a good standard of care) [84,85]. Further research is needed to establish the effect of owner attachment on their rat care practices, and is another influence to be aware of when designing future research.

### 4.5. Participant and Rat Contextual Background

It was clear from what owners said about their rats that they felt them to be highly characterful with their own individual personalities. As this study was qualitative in nature and relied on owner reports, it is important to consider how these anthropomorphic interpretations may influence owners’ perceptions of their rats and the behaviours they reported. Furthermore, the feelings of love that owners expressed towards their rats was evident. Similarly, rat owners have previously reported (in a questionnaire) that they agreed that rats are friendly, loveable, intelligent and curious [37].

An Interpretative Phenomenological Analysis by Dickson et al., (2019) found that owners of cats, dogs and rabbits reported strong and affectionate relationships with their pets. Participants talked about the unconditional love they experienced from their pets, with many describing themselves as their pets’ “mummy” [79]. Participants in this study also reported strong and affectionate relationships with their rats. The terminology used by pet owners is often dependent on social factors, such as whether the individual is conversing more informally (e.g., ‘pet-parent’, ‘mum’ or ‘dad’ to the pet or ‘baby’ with family and friends) or formally (e.g., ‘owner’ and ‘pet’ with coworkers) [81]. As many of the participants in the current study used the former terminology when discussing their rats, it is likely that the researcher conducting the interviews (CW) was able to establish a good rapport with participants, allowing for more detailed information to be collected [57].

Previous work on pet-parenting has suggested that having children is negatively associated with the strength of bond and investment in the pet [81,83,85]. Despite concerns raised in the previous Section ‘4.4 *Owner Practices*’ about the potential implications of this anthropomorphic framing, Volsche (2021) suggested that nonparents invested in their pets in ways that met their species-specific needs [85]. The present study did not collect information on whether the participants had children so no comparison can be made. More research is clearly needed to investigate specifically if and how pet-parenting may affect the housing and husbandry of pet rats. However, it seems likely based on the language used by participants in this study that pet-parenting style and anthropomorphism may influence these management practices as well as how participants viewed different rat interactions. It is therefore important to take the findings of this study alongside the understanding that participants’ perceptions may be shaped by underlying pet-parenting narratives, that can both enhance engagement with animals and introduce bias into behavioural assessments.

Furthermore, in Robin et al., (2017), pet rat owners talked about themselves and their rats receiving stigma because pet rats are not generally socially acceptable [86]. This is a sentiment that appears to be shared in this study, although this topic was not discussed as deeply in this study as in Robin et al., (2017) [86].

There remains a lack of academic research into the human–pet rat bond, with Schneidewind (2024)’s work a notable exception [37]. There is a risk of poorer welfare with less socially valued companion animals, however, our findings suggest that owners form strong bonds with their pet rats, challenging this assumption and the negative stereotypes surrounding pet rats. These findings are important because they demonstrate that this often-overlooked companion animal is capable of eliciting strong attachments from their owners. Recognising this bond has practical welfare implications as it suggests that owners may be motivated to provide higher standards of care, and it challenges common misconceptions that rats are lesser pets. More broadly, these results extend the field of human–animal interaction beyond traditional species, opening new avenues of research into how such bonds affect rat welfare and human wellbeing.

### 4.6. Owner Narratives and Shared Understandings

Due to the nature of the open-interviews, participants sharing stories was common. Where these stories did not fit easily into other themes (often due to the level of detail and multiple themes threaded throughout), these were given their own theme: Owner Narratives and Shared Understandings. These narratives help to contextualise many of the themes discussed above.

The complexity of many social behaviours and dynamics were highlighted, suggesting that while the context of behaviours within a situation is an important consideration, so is the background of the rats in question. Many of the participants in this study owned rats that they had rescued, which may account for why this consideration came up. Thumpkin et al., (2024) reported that owners identified building trust with rescue dogs took time, with some even reporting that their dogs’ behaviour changed as they settled into their new home [87]. While the dogs’ background was not explicitly mentioned, it may have played a part in how they settled into their new home. Further research is needed to understand how animals’ backgrounds shape their behaviours and experiences within pet environments. The rat owner population may be a good starting point for this due to rat owner willingness to share their experience of rat ownership, including rats with more unique backgrounds.

The expression of grief in rats was also highlighted by owners, particularly with regard to the importance of social bonds and the impact of losing those bonds. It has previously been shown that rats bury their dead and olfactory signals (e.g., the accumulation of polyamines in the deceased body giving it a dead smell) have been suggested as a reason for this [88]. Mice have been observed performing behaviours thought to be aimed at resuscitating unresponsive familiar partners [89]. These behaviours start off gently (e.g., sniffing and grooming) but become more forceful (e.g., biting the partner’s mouth and pulling their tongue out) when the unresponsive partner continues to be unresponsive. These behaviours are unlikely to be due to curiosity or an attempt to initiate a social interaction as they were strongly influenced by familiarity, suggesting the motivation behind these behaviours may be to revive the partner [89]. In contrast to this, necrophobic behaviour has been observed in mice, although whether or not the necrophobic mice were familiar with the deceased ones is not stated [90].

Cat and dog owners have reported behavioural changes following the loss of an animal companion [42,91,92]. Statistically significant changes (both behavioural—e.g., playing, sleeping, eating; and emotional—e.g., exhibiting fearfulness) in surviving pet dogs after the loss of a conspecific have been reported by owners [91]. Therefore, it seems reasonable to suggest that rat behaviour may change after the death of a conspecific due to grief. However, grief cannot be confirmed as the reason behind these changes and further research is required to establish any causal relationships. Greene and Vonk (2024) found that owners with a higher level of attachment to their cats reported more instances of attention-seeking behaviour in their cats after the death of another pet, which may reflect anthropomorphism of the cats rather than a grief-like response [92].

One possible explanation for necrophobic behaviour is that the presence of a dead conspecific may pose a predation risk to the remaining individuals. It may therefore be possible that behavioural changes observed, especially in response to being shown the body of their deceased conspecific, may be due to an intrinsic response to protect themselves, rather than to a more anthropomorphic framing of grief. It would be useful to assess these changes using observations to determine whether these reports are likely to be a product of anthropomorphism, or an emotional response to the death of a conspecific.

### 4.7. Owner Research Interests

In a study investigating pet owners’ perceptions of information exchange about companion animals, many owners acknowledged that sources of information they used were not reputable [93]. Rat owners in this study also acknowledged this, suggesting that an established and reputable source of rat care information does not exist thus far.

One owner in the present study mentioned that they would like to see some research into pet rat introductions using cortisol as a measure of stress. While in most mammals, cortisol is considered the primary measure of endogenous adrenal steroid, this is not the case in rats, where corticosterone is a more relevant biomarker for stress [94]. For this reason, we would consider corticosterone (measured through blood, saliva, faecal, or hair samples) to be a more appropriate measurement. While salivary or serum corticosterone analysis would likely be the best way to measure acute stress during an introduction, obtaining salivary or serum corticosterone samples from rats may be difficult and invasive in practice. LaFollette et al., (2018) collected faecal corticosterone (along with vocalisations and behavioural observations) of rats in pet stores [95]. Despite not being a reliable measure of stress in the moment (e.g., faecal corticosterone would not be able to indicate whether rats immediately became more stressed upon the introduction of a new, unfamiliar individual), faecal corticosterone could be used as a physiological measure of rat stress over a longer period of time (e.g., measures could be taken one week before the introduction, one week into the introduction and one month after the introduction).

Behavioural observations are a commonly used and reliable measure in laboratory rats that could be employed in future research looking at rat introductions [96,97,98]. Behavioural observations could make use of already existing ethograms (e.g., Schweinfurth (2020)’s ethogram of social behaviours [3]) and record the frequency of affiliative (or socio-positive) behaviours and agonistic (or socio-negative) behaviours during introductions. Vocalizations are another potentially useful and uninvasive measure [95,99].

This exploratory study has opened many potential future avenues for pet rat research encompassing rat–rat social relationships (including research into pet rat introductions), human-rat relationships, and owner practices (e.g., how conflict between rats is managed). An immediate follow-on to the present study would be to explore how housing configuration (e.g., pair vs. group housed rats) and sex influence the social dynamics and behaviours of pet rats. Future studies could feasibly incorporate owner-led sampling kits, behavioural video recording and longitudinal tracking of welfare indicators. Challenges include ensuring sample integrity, training owners to follow protocols and maintaining engagement over time. Leveraging an enthusiastic owner community will be imperative to overcome these challenges.

Rat owners in this study were enthusiastic about research on pet rats being conducted. A survey on veterinary biobanking found that 91% of participants contributed because they believed it would increase the chances of a cure for future animals with the same condition, 84% contributed because their pet, family or other pets could benefit from the research, and 80% took part because they wanted to contribute to future research [100]. Rat owners in this study did not give a reason for their motivation to take part in research, but did express how happy they were that research was being conducted.

It could be very useful to employ the help of rat owners in determining future research directions for pet rat research—both in the recruitment of participants, but also to aid in the identification of opportunities to improve the welfare of pet rats. However, as is often the case in self-selected volunteers, it is likely that more engaged rat owners will come forward to participate. More ‘typical’ rat owners (e.g., people without prior experience of small animals or owners who view rats primarily as low-maintenance pets), in which welfare standards may often be lower, are likely to be more difficult to reach. To mitigate this bias, recruitment should take place through a diverse range of channels (e.g., online communities, pet stores, veterinary clinics, etc.).

There is also potential to align methodologies with those used in laboratory rat welfare research, enabling cross-context comparisons and facilitating knowledge exchange. These authors see potential for a mutual knowledge exchange between the pet rat community (both researchers and owners) and laboratory rat specialists with a view to improve both the understanding and the welfare of all the rats. For example, through collaborative projects to adapt laboratory enrichment designs for pet settings and vice versa, co-developed owner education materials, and shared workshops linking researchers, laboratory welfare professionals, veterinarians, and owners. Such cross-sector engagement could broaden the evidence base available to pet rat keepers while also offering insights into methods of improving laboratory rat welfare.

### 4.8. Reflective Thematic Analysis in the Scientific Context

Using owner reports to study companion animal social behaviour provides valuable insights that would otherwise be difficult to capture in controlled laboratory settings. Owners observe their animals across a wide range of contexts and over extended timeframes, allowing for the identification of subtle or rare behaviours. When analysed thematically, such reports can highlight common patterns in owner perceptions, contributing to an overall richer understanding of the behavioural repertoire. However, it is important to note that the reports of enthusiastic pet owners might not always provide fully reliable information. For example, some participants may have emphasised some (e.g., more positive) aspects of their rats’ social behaviours, while underreporting others. Furthermore, their interpretations of rat social behaviours and relationships are inevitably subjective and therefore lack the rigor and clarity of objective, laboratory-focused investigations. It is well known that humans have a strong tendency to anthropomorphise animals (particularly pet animals [101]), and this is likely to be the case here. Therefore, while the observations that can be obtained from pet owners are certainly valuable, and less likely to be constrained by experimenter knowledge, hypotheses, etc., their interpretations and inferences will need to be assessed with care. They can, however, be used as a starting point for more systematic observational studies or experimental designs that seek to verify and expand upon the patterns identified in owner reports. For example, Samet et al., (2022) used a systematic literature review to assess which domains of the human–dog bond are already well-covered and where there may be areas to develop. The findings of this review were then paired with owner interviews to identify themes that could be used to improve current assessment tools for the human–dog bond [102].

Exploratory studies into pet rat care and its implications on welfare have been conducted by Neville et al., (2021), Neville et al., (2022), and Schneidewind et al., (2024) [10,36,37]. The present study builds on this work, focussing on the social aspect of welfare. As demonstrated throughout, much is already known about the social behaviours and relationships of rats in other contexts; therefore, it was felt that a good first step in researching this social element of pet rats would be to gather insights from those that spend the most time with them: their owners. Of course, the absence of direct behavioural verification of these owner accounts means this reflective thematic analysis should be interpreted with caution and ideally complemented in the future by some of the observational and experimental designs outlined in the previous section.

### 4.9. Study Limitations

Reflective thematic analysis is inherently interpretive, and findings are shaped by the researcher’s position, values and reflective engagement with the data. The researcher engaging with the dataset actively and reflectively in shaping the final product is an important part of a reflective thematic analysis [103]. While this is a strength in enabling depth, it also introduces the limitation of subjectivity. Transcripts were used to record verbatim what was said rather than relying on notes made by the researcher at the time of the interview, along with repeatedly reviewing the content of the transcripts, and regular consultation between this paper’s authors, to overcome as much of this limitation as possible [46,57]. Furthermore, the assumptions and positionings of the researcher are a part of reflective thematic analysis [46]. The researcher conducting the analysis (CW) has owned pet rats for twelve years and is an active member of the online pet rat community. This allowed for valuable insights and rapport between the interviewer and interviewees. The researcher was already familiar with some aspects of rat social behaviour and dynamics but was also aware of the limited research base and lack of consensus regarding how these behaviours are interpreted in pet contexts. The researcher’s awareness of their own positionality in this research was important and a summary of the researcher’s reflections have been detailed in Supplementary Materials File S3.

As with many qualitative methods, reflective thematic analysis prioritises depth over breadth, meaning that findings are context specific and cannot always be generalised to the wider population in the same way that quantitative data can be [46]. A total of 23 participants were interviewed in this study and data saturation was achieved following the data saturation checklist proposed by Ahmed (2025) [51]. Six to twelve individuals is generally believed to be a sufficient sample size in this type of study [52]. Previous research of a similar nature to this study have four to 16 participants (e.g., Hale, et al., (2023) had four participants that took part in semi-structured interviews to examine their quality of life tool [104], Bacon et al., (2021) conducted their structured interviews with eight Chinese and eight European zoo staff [105] and Lea et al., (2025) had eight undergraduate students take part in their semi-structured interviews [106]. Furthermore, the only other thematic analysis with pet rat owners as participants known to these authors had seven rat owners who took part in the semi-structured interviews [86]. In this study, 88% of the behaviours and 67% of the types of social relationships in the entire dataset had been identified after the first eight open interviews (the point at which data collection was paused and data saturation reviewed before it ultimately continued to achieve data saturation). Despite this data saturation, it is still not possible to generalise these findings to the wider population. This exploratory study is a useful starting point but further research, particularly around validating these findings quantitatively is needed.

Participants in this study were recruited via a social media call in UK-based pet rat Facebook groups asking for participants for an interview about pet rat social relationships. As identified in previous pet rat research [36,37], there is likely to be a degree of participation bias in which owners chose to take part. This may have led to an over-representation of highly engaged rat owners. While potentially limiting in terms of not including a more broad overview of owner perceptions that could be generalised to the general rat-owning population, this participation bias may have nevertheless led to a more detailed exploration of pet rat relationships and behaviours.

## 5. Conclusions

This study aimed to explore the social relationships and behaviours of pet rats, as seen through the eyes of their owners. To do this, open-ended interviews with owners and a reflective thematic analysis were undertaken, and seven main themes were generated: Social Behaviours, Social Life and Group Dynamics, Introducing New Rats and Repairing Social Bonds, Owner Practices, Participant and Rat Contextual Background, Owner Narratives and Shared Understandings, and Owner Research Interests. Rats sleeping in close proximity, playing and allogrooming were identified as positive (affiliative) behaviours, with owners also reporting neutral and ambiguous types of grooming (dominant grooming and overgrooming, respectively). A number of behaviours were reported to be warning signs of fighting, which was identified as negative (agonistic). Some owners also identified other, ambiguous behaviours that may convey extreme emotions and whose meaning is likely to be context specific. Owners commonly described rat groups as having a clear leader, or ‘alpha’ rat, and generally believed groups to get along. However, key questions emerge about the welfare implications of co-housing rats that do not get along. This study includes the first academic report of pet rat introductions, introducing the ‘Carrier Method’, as well as the expression “heart rat”, used to convey the special bond owners feel towards certain rats. Finally, a potential avenue for future relationships between researchers and the pet rat community has been highlighted by the enthusiasm expressed by owners in this study. The findings discussed in this paper help to further our knowledge of the social relationships and behaviours of pet rats, which it may be possible to extend to other rat populations (e.g., laboratory rats). In particular, these findings shed light on how owners manage social dynamics within their rat groups and included the first scientific report of rat introductions. Further work to gather a wider sample and to capture these social behaviours and dynamics in a more standardized manner through observational studies is required.

## Figures and Tables

**Table 1:** Information about the participants’ ages, gender, and the total number of rats they had owned in their lifetime.

Frequency	**Age Category**					
	**18–24 yrs**	**25–34 yrs**	**35–44 yrs**		**45–54 yrs**	**55–64 yrs**
	1	11	7		2	2
	**Gender**					
	**Woman**			**Man**		
	21			2		
	**Total number of rats owned in lifetime**					
	**2**	**3–5**	**6–10**	**11–20**	**21–49**	**50–99**
	2	1	5	4	7	4

**Table 2:** Demographic information about the participants’ currently owned rats.

Participant Number	Number of Rat Groups Owned	Total Number of Rats Owned	Total Number of Male Rats Owned	Total Number of Female Rats Owned
O1	2	13	2	11
O2	1	3	3	0
O3	1	4	0	4
O4	1	11	11	0
O5	1	3	0	3
O6	1	5	0	5
O7	3	15	14	1
O8	1	4	4	0
O9	2	7	7	0
O10	2	6	6	0
O11	1	2	2	0
O12	2	12	12	0
O13	1	5	5	0
O14	3	13	13	0
O15	1	2	2	0
O16	1	6	6	0
O17	1	4	0	4
O18	2	14	8	6
O19	2	6	0	6
O20	1	3	0	3
O21	1	5	5	0
O22	1	5	0	5
O23	1	8	0	8

**Table 3:** Table detailing owner reported positive, negative and ambiguous behaviours.

Subtheme	Behaviour
Positive	Grooming companions (or allogrooming)
	Sleeping in close proximity
	Prancing
	Playing
	Chasing Following another rat
	Popcorning
	Taking cues from each other
	Hopping
Negative	Tussles/Scraps/Squabbles
	Tensing up
	Sidling/Going on side
	Fur puffed up
	Rat ball
	Fighting
	Teeth baring
	Huffing
	Scratches/Bites
	Placing paw on rat to stop them
	Pushing
	Kicking
	Pulling fur
	Chasing
	Humping
	Boxing
	Hunched posture
	Shuffling
	Screaming
	Pinning
	Stealing
	Moving around each other
	Cornering
Ambiguous	Squeaking Ear twitching
	Vibrating
	Dominant grooming
	Overgrooming
	Bruxing
	Boggling
	Tail wagging
	Genital inspection

## Data Availability

The original contributions presented in this study are included in the article/Supplementary Materials. Further inquiries can be directed to the corresponding author(s).

## Supplementary Materials

The following supporting information can be downloaded at: https://www.mdpi.com/article/10.3390/ani15172579/s1, Figure S1: Initial thematic map produced from open interviews. Dark blue ovals indicate a theme, blue rounded squares indicate subthemes, turquoise ovals indicate subtopics, grey lines indicate a link to a subtheme and broken purple lines show relationships between topics; Figure S2: Final thematic map produced from open interviews. Dark blue ovals indicate a theme, blue rounded squares indicate subthemes, grey lines indicate a link to a subtheme and broken purple lines show relationships between topics; Table S1: Table detailing the themes and their definitions; Table S2: Table detailing how the themes were refined; File S3: Summary Reflection Diary.

## Appendix A

**Owner Demographic Survey**

To which age category do you belong?

○ 18–24
○ 25–34
○ 35–44
○ 45–54
○ 55–64
○ 65+

What is your gender?

○ Man
○ Woman
○ Non-binary
○ Prefer not to say
○ Other

In your lifetime, how many pet rats have you had in total?

Free text answer

## Appendix B

**Semi-Structured Interview Topic Guide**

*Note: Questions are likely to develop and change throughout the process of interviews, as new themes present themselves and require exploration.*

**Section 1: Basic information about your rats ***

1. How many rats do you have at the moment?
2. Are they housed in different groups (i.e., cages or enclosures)?
3. What are their names?
4. Are they males or females?
5. Are any of them neutered? If so, what was the reason for this?
6. How are your rats housed?
7. What type of cage(s)/enclosure(s) do they have?
8. What’s in the cage? (i.e., nesting area, dig box, things to climb on, gnawing blocks, etc.)
9. What substrate/bedding do you use?
10. How do you feed your rats? And how often?
11. Do your rats routinely spend time outside of their cage? (i.e., apart from one another?)

**Section 2: Your rats’ relationships (and the factors that determine this)** *These questions will be asked for each group of rats*

12. Do you think your rats get along? Can you describe the relationships within this group of rats? Do you think they have established a social hierarchy i.e., Would you say that any particular rat is ‘the boss’? What makes you say this?

Does this change?

13. Are there any rats within the group that you would say are ‘friends’? Why do you think this?
14. Are there any rats within the group that you think do not get on very well? Why’s this?

**Section 3: What factors make you think this?** These questions will be asked for each group of rats

15. Can you give me any examples of positive/friendly social interactions within the group?
16. Can you give me any examples of negative/unfriendly social interactions within the group?
17. Are there any specific behaviours or patterns of interaction that stand out to you?
18. How important do you think it is that rats in a group get along?

**Section 4: Do the social relationships change and what do you think causes this?**

19. Have you seen any changes in relationships within the group over time? What do you think caused this?

*** (Analysis of these questions is beyond the scope of this paper)**

## Appendix C

A number of stories provided by owners that give more context to points made throughout the main text have been provided below.

*“There’s one called [Rat 1], and she’s been the hardest one. She was the one we got from the rescue place and she is... We got the three, [Rat 1], [Rat 2] and [Rat 3] and she’s the hardest one. We’ve spending a lot of time like handling with.. she is very timid. And has to do her own thing. But she’s in the pile still, but she does like to take herself off a lot, but she’s in the pile, which is good. She was the one I was worried about when we brought the new babies in, but she’s accepted them. She’s doing really well with them, which is really good.” [O18]*

*“Well, he didn’t even know how to rat really, because he literally as soon as I put him in with my rats, like even in a small hamster cage, instantly he just dug a moat with all the substrate around him. So everybody was over there. And he was in this corner with like a line that… he’d literally dug a line around himself and was like, had his teeth out at everybody. And it was like I thought this I do not know how I’m going to do this because he’s just... It’s a lot, but we got there. It took a lot. It took a few days, but we, you know, in a very small space. But we got there with him. He’s he’s always going to be weird, but it’s just how he is, unfortunately, there’s nothing you can do about it.” [O4]*

These stories add a new level of context to consider when it comes to the dynamics of rat groups: the background of the rats involved.
