# Supplementary material for: Through Human Eyes: Owner Insights into the Social Relationships of Pet Rats
Source: Animals (Basel). 2025 Sep 2;15(17):2579. doi: 10.3390/ani15172579 (PMC12427543; doi:10.3390/ani15172579)
Supplement: Supplementary file 1 [file animals-15-02579-s001.zip › Supplementary Material File S1.pdf]

Supplementary Material File S1

An initial thematic map (Figure S1) was produced of the 16 themes.

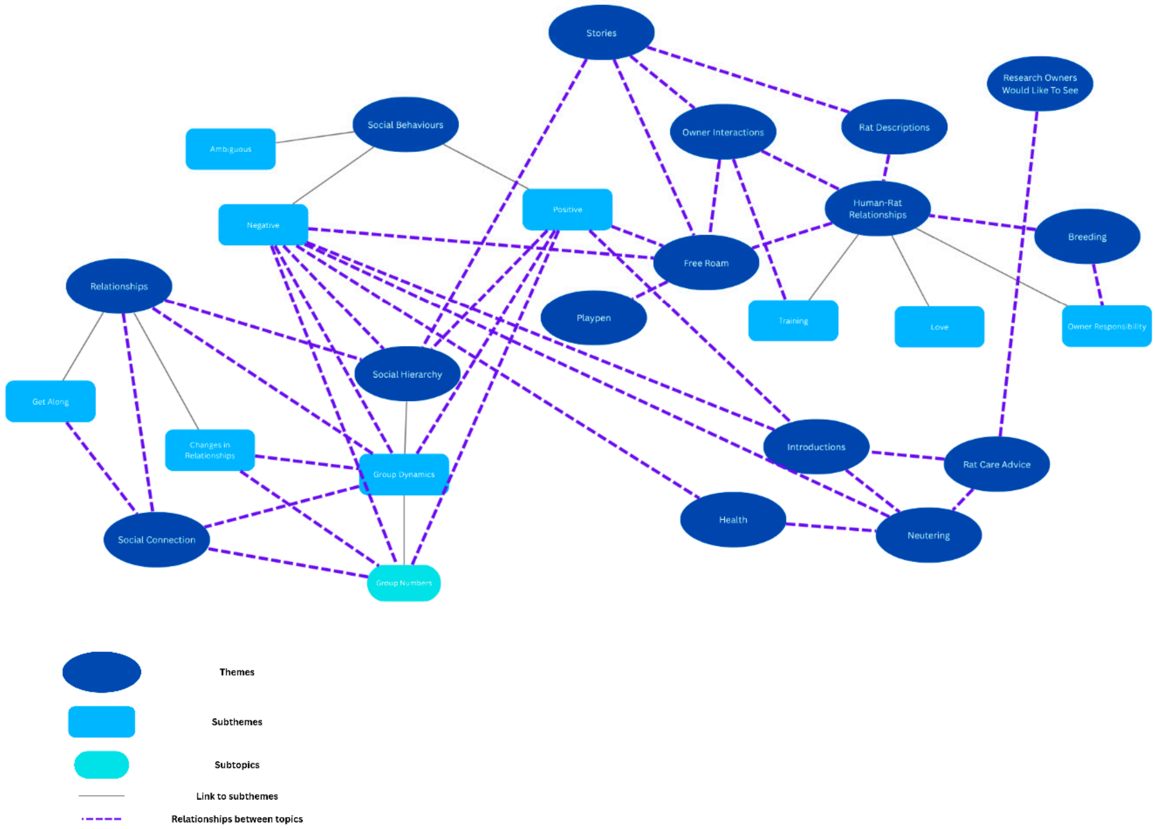

Figure S1. Initial thematic map produced from open interviews. Dark blue ovals indicate a theme, blue rounded squares indicate subthemes, turquoise ovals indicate subtopics, grey lines indicate a link to a subtheme and broken purple lines show relationships between topics.

Table S1 outlines the definitions of these themes and subthemes.

Table S1: Table detailing the themes and their definitions.

Theme	Subtheme	Sub-subtheme	Definition
Introductions			Any mention of rat introductions or bonding individuals
Rat Descriptions			How owners describe their pet rats
Relationships			Owner descriptions of the relationships

			within their rat groups
	Get Along		Owner comments on how important they feel it is for rats in a group to get along with one another
	Changes In Relationship		Comments about how the relationships within the rat group changed over time
Social Hierarchy			Owner comments on the social hierarchies of their rat groups
	Group Dynamics		Comments relating to more general dynamics within rat groups
		Group Numbers	Owner comments about the ideal number of rats in a group
Behaviours			Any mention of a behaviour
	Positive		Behaviours that owners identified as positive
	Negative		Behaviours that owners identified as negative
	Ambiguous		Behaviours that owners weren't sure about or found odd
Owner Interactions			Comments about how owners interact with their rats and vice-versa
Human-Rat Relationships			Owner comments about their own relationships with their rats
	Love		Relating to positive relationships

			between the owner and rats
	Training		Any mention of rat training
	Owner Responsibility		Owner comments about their responsibility or sense of duty towards their rats
Breeding			Any mention of rat breeding
Health			Owner comments made about their rats' health
Stories			Examples and stories given by owners
Neutering			Any mention of a rat being neutered
Social Connection			Owner comments about the importance of social connections in rats
Research Owners Would Like To See			Comments made by participants on what research on pet rats they would like to see done
Rat Care Advice			Comments made by participants on current pet rat care advice
Playpen			Any mention of a play pen or enclosure for rats to free roam in
Free Roam			Any mention of free roam time
