# Supplementary material for: Through Human Eyes: Owner Insights into the Social Relationships of Pet Rats
Source: Animals (Basel). 2025 Sep 2;15(17):2579. doi: 10.3390/ani15172579 (PMC12427543; doi:10.3390/ani15172579)
Supplement: Supplementary file 1 [file animals-15-02579-s001.zip › Supplementary Material File S2.pdf]

Supplementary Material File S2

Themes were refined by combining similar themes together and a final thematic map (Figure S2) was produced.

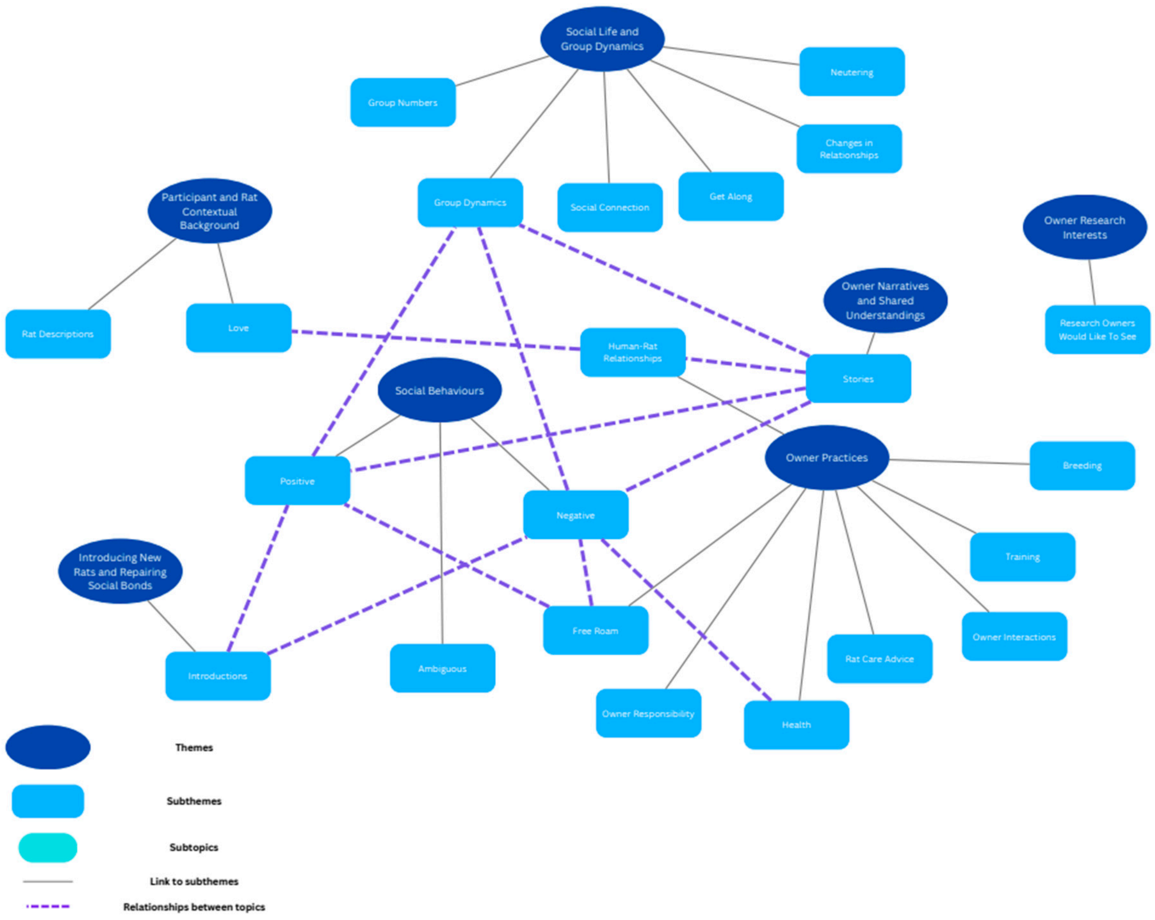

Figure S2. Final thematic map produced from open interviews. Dark blue ovals indicate a theme, blue rounded squares indicate subthemes, grey lines indicate a link to a subtheme and broken purple lines show relationships between topics.

Details of refinement are in Table S2.

Table S2: Table detailing how the themes were refined.

**Refined Theme**	**How it was generated**
Social Behaviours	The initial theme of *behaviours* and subthemes of *positive*, *negative* and *ambiguous* were combined

Social Life and Group Dynamics	The initial themes of *social hierarchy*, *relationships* and *social connection* (and their subthemes), along with the theme of *neutering* were combined
Introducing New Rats and Repairing Social Bonds	This is the new name for the theme of *introductions*
Owner Practices	The initial themes of *breeding*, *owner interactions*, *rat care advice*, *health*, *free roam*, *playpen* and *human-rat relationships* (and their subthemes, except for the subtheme of *love*) were combined
Participant and Rat Contextual Background	The theme of *rat descriptions* and the subtheme of *love* were combined
Owner Narratives and Shared Understandings	This is the new name for the theme of *stories*
Owner Research Interests	This is the new name for the theme of *research owners would like to see*
