# Supplementary material for: Through Human Eyes: Owner Insights into the Social Relationships of Pet Rats
Source: Animals (Basel). 2025 Sep 2;15(17):2579. doi: 10.3390/ani15172579 (PMC12427543; doi:10.3390/ani15172579)
Supplement: Supplementary file 1 [file animals-15-02579-s001.zip › Supplementary Material File S3.pdf]

Supplementary Material File S3

Summary Reflection Diary

Social Behaviours

As a researcher, I was surprised at how sleeping together was unanimously considered so positively. It really stood out to me as a clear indicator to owners of how well their rats were getting along. I have always seen this as just something that they do, but it does make sense that sleeping together would be a positive thing. I find the nuance in how rats are sleeping together particularly interesting as well: there appears to be a distinction between sleeping together and sleeping together in a relaxed manner, like overlapping. I feel that this distinction is particularly noteworthy.

I expected owners to identify rats grooming each other as a positive behaviour so was surprised about the level of ambiguity with this behaviour. It seems again that a distinction in how this behaviour is being performed is used to identify its valence.

I expected playing to be classed as a positive behaviour.

Negative behaviours mentioned by owners generally lined up with my own expectations of agonistic behaviours.

Chasing is an interesting behaviour – quite often it is mentioned positively in the context of playing, but it is also more often mentioned negatively in the context of fighting.

As a researcher, the explanation that owners notice more negative behaviours than positive ones because the positive ones are quieter and less obvious makes a lot of sense to me. It also seems to fit well with the data that has been collected: while owners are able to report some positive behaviours, they reported many more negative ones.

As a researcher, I find the distinction in grooming types to be particularly fascinating. There is normal allogrooming which is identified as positive, overgrooming which is identified by some owners as positive and others negative, and dominant grooming which is not identified as positive or negative – just a

normal aspect of rat groups. Perhaps more research into grooming types could be useful?

I really like the mention of certain behaviours (e.g. boggling, bruxing, tail wagging) being indicators of extreme emotions. My experience of bruxing and boggling from the perspective of a pet owner is that they are generally positive, with the caveat that they can also be indicators of pain. My experience of tail wagging has been in both the contexts of positive anticipation and as a fear response. Boggling has only recently been described in the scientific literature (Neville et al., (2022)) so further research is definitely needed to understand the contexts and meanings of these behaviours, but owner reports in this study generally fit with what I would have expected.

Social Life and Group Dynamics

Upon reflection, owners frequently describing their rat groups as having a dominant individual or 'alpha' rat was to be expected given the questioning about social hierarchies and whether or not there was a 'boss rat'. However, as a researcher, I was surprised by how quickly and consistently owners reported these terms.

Interestingly, owners also said that more cohesive groups tended to be the ones where they could not tell who was in charge, suggesting that where owners were able to describe a hierarchy in the group, the group may not have been particularly cohesive.

I find the following excerpt particularly noteworthy:

"They're all buddies really, to different extents I think." [O1]

It reflects a nuanced perspective among rat owners: while there is a general belief that rats get along well, there is also an awareness that this is not universally true. I think this is particularly important to note. In the rat groups, I often see it's advised that people do not keep rats on their own (as they are social animals), but from a welfare perspective, are there scenarios where being housed singly would be more beneficial for the rat in question? What are the welfare implications of housing rats that do not get along together?

Introducing New Rats and Repairing Social Bonds

Interesting that all owners who mentioned introductions used the carrier method. From my experience in the pet rat community, this is this the most common method of rat introductions. Is it the best way though? I am also aware of other methods but to my knowledge, there does not appear to be any evidence showing what the best method, from a welfare perspective, might be.

Owner Practices

As a researcher, I particularly love the term 'heart rat' as it conveys so well the genuineness of the bond that owners have spoken about.

The bond the owners talk about is really powerful. As a researcher, I feel that the depth of the bond owners feel for their rats deserves to be highlighted.

Participant and Rat Contextual Background

I really like the way that owners have conveyed the personality of their rats over and over. It is very clear how much they love them. As a researcher, I found myself reflecting on how these personalities may play a part in the context of the responses I received about social relationships. How does this (anthropomorphic?) view colour the way owners see their rats, and subsequently what they have reported?

I also want to highlight the following excerpt:

"…they are all proper little characters and people don't think that they are, but they really are little characters…" [O18]

As a researcher, I found myself reflecting on the undertone from this excerpt that many people do not see pet rats in the same way that their owners do. There was a feeling with this excerpt that rats are often misunderstood, but not by their owners. This is an experience that I can personally relate to and feel it could be useful to highlight.

As a researcher, I felt that it was important to view the final dataset through the lens of affection that the owners so frequently conveyed.

Owner Narratives and Shared Understandings

There are some really nice examples that contextualise and illustrate what has already been placed elsewhere – excerpts that are too full of codes to be placed in other sections, but still noteworthy.

Owner Research Interests

As a researcher, I feel that there not being very much established rat care advice out there is a very significant point in the context of existing literature. While considerable research has focused on laboratory rats, there remains a notable gap in understanding pet rats, which are comparatively underrepresented. There is also a lot of rat care information out there for pet owners but it is not usually known where that advice comes from and whether or not it has any scientific bearing.
